# The global effect of follicle-stimulating hormone and tumour necrosis factor α on gene expression in cultured bovine ovarian granulosa cells

**DOI:** 10.1186/1471-2164-15-72

**Published:** 2014-01-28

**Authors:** Claire Glister, Nicholas Hatzirodos, Katja Hummitzsch, Philip G Knight, Raymond J Rodgers

**Affiliations:** 1 School of Biological Sciences, University of Reading, Hopkins Building, Reading RG6 6UB Whiteknights, UK; 2 Discipline of Obstetrics and Gynaecology, School of Paediatrics and Reproductive Health, Robinson Institute, University of Adelaide, 5005 Adelaide, SA, Australia

**Keywords:** Ovary, Microarray analysis, Bovine granulosa cells, Follicles

## Abstract

**Background:**

Oocytes mature in ovarian follicles surrounded by granulosa cells. During follicle growth, granulosa cells replicate and secrete hormones, particularly steroids close to ovulation. However, most follicles cease growing and undergo atresia or regression instead of ovulating. To investigate the effects of stimulatory (follicle-stimulating hormone; FSH) and inhibitory (tumour necrosis factor alpha; TNFα) factors on the granulosa cell transcriptome, bovine ovaries were obtained from a local abattoir and pools of granulosa cells were cultured *in vitro* for six days under defined serum-free conditions with treatments present on days 3–6. Initially dose–response experiments (n = 4) were performed to determine the optimal concentrations of FSH (0.33 ng/ml) and TNFα (10 ng/ml) to be used for the microarray experiments. For array experiments cells were cultured under control conditions, with FSH, with TNFα, or with FSH plus TNFα (n = 4 per group) and RNA was harvested for microarray analyses.

**Results:**

Statistical analysis showed primary clustering of the arrays into two groups, control/FSH and TNFα/TNFα plus FSH. The effect of TNFα on gene expression dominated that of FSH, with substantially more genes differentially regulated, and the pathways and genes regulated by TNFα being similar to those of FSH plus TNFα treatment. TNFα treatment reduced the endocrine activity of granulosa cells with reductions in expression of *FST*, *INHA*, *INBA* and *AMH.* The top-ranked canonical pathways and GO biological terms for the TNFα treatments included antigen presentation, inflammatory response and other pathways indicative of innate immune function and fibrosis. The two most significant networks also reflect this, containing molecules which are present in the canonical pathways of hepatic fibrosis/hepatic stellate cell activation and transforming growth factor β signalling, and these were up regulated. Upstream regulator analyses also predicted TNF, interferons γ and β1 and interleukin 1β.

**Conclusions:**

*In vitro*, the transcriptome of granulosa cells responded minimally to FSH compared with the response to TNFα. The response to TNFα indicated an active process akin to tissue remodelling as would occur upon atresia. Additionally there was reduction in endocrine function and induction of an inflammatory response to TNFα that displays features similar to immune cells.

## Background

An ovarian primordial follicle is composed of an inactive oocyte surrounded by granulosa cells all enclosed by a basal lamina. The granulosa cells of the ovarian follicle support and nurture the oocyte, and secrete oestrogens which are necessary for normal reproductive function. In mammals, the latter stage of follicle development can involve an approximate hundred fold increase in diameter, 21 doublings of granulosa cell numbers [1] and formation of a fluid-filled antrum [2]. In cattle, the growth of follicles is tightly regulated, since two or three groups or waves of follicles emerge from a pool of follicles larger than 5 mm in diameter during each oestrous cycle [3,4]. In these waves, follicles continue to enlarge over several days until one follicle grows faster and larger than the others and hence gains ‘dominance’ [5,6]. This deviation in size occurs when the follicles are around 7–8 mm in diameter [7]. These processes of follicular growth occur largely due to the stimulatory influence of FSH, through its receptor localised exclusively to the granulosa cells, though other factors produced locally, for example Growth Differentiation Factor (GDF)-9 [8] and Bone Morphogenetic Protein (BMP)-15 from the oocyte [9], are also involved.

Instead of one primordial follicle growing to ovulatory size and then ovulating, many follicles commence growing during the course of the cycle. Most of these growing follicles become atretic, resulting in cows and humans, in only one or occasionally two follicles ovulating each cycle. The highest rates of atresia in follicular development occur around the time of antrum formation. It has been shown that the atretic process begins with cell death in the membrana granulosa initially by an apoptotic process [10]. Generally, apoptosis may be instigated intracellularly by cytotoxic stress, possibly due to free radicals or calcium influx [11] which cause mitochondrial changes that eventually also lead to caspase activation. Apoptosis can be initiated externally to the cell by the binding of ‘death’ ligands such as Fas ligand, tumour necrosis factor α (TNFα) or TRAIL to specific receptors [12]. In follicular atresia it is unlikely that cell death occurs on a cell-by-cell basis because numerous pyknotic nuclei are observed during atresia [10]. Therefore it is probable that atresia is initiated by either the presence or absence of a particular external signal(s). TNFα can initiate apoptosis in granulosa cells [13,14]. The expression of TNFα receptors on granulosa and theca cells has been shown to be increased in atretic follicles when compared with healthy small or preovulatory follicles [15]. Studies on atretic follicles so far have shown that many of the genes/pathways involved are common to those stimulated by TNFα, as recently reviewed by Matsuda *et al.*[16].

Investigation of the effects of various agents on granulosa *in vitro* is dependent on the follicle stage at which the cells were isolated and the composition of the culture medium. It has been demonstrated that granulosa from small antral follicles are more responsive to FSH in serum-free culture and are capable of increasing oestradiol production over a six day period [17,18]. This is an important consideration for studying granulosa cells as they have a propensity to differentiate into granulosa-lutein cells in a process called luteinisation, if cultured in serum-supplemented medium [19]. Such cells are completely unresponsive to FSH. A previous study showed that TNFα was able to block the effects of FSH in serum-free culture of rat granulosa cells [20]. We were interested in the effect of FSH and TNFα on steroid production and global gene expression in bovine granulosa cells to help elucidate the mechanisms of action of these compounds at the transcriptional level. Interest in the action of pro-inflammatory signals like TNFα has been augmented by recent reports that ovarian granulosa cells of cattle and other species fulfil an innate immunity role, being capable of detecting and responding to bacterial pathogens [21]. We therefore cultured granulosa cells from small bovine follicles with or without FSH and TNFα, alone and in combination, assayed steroid hormone production by immunoassay and conducted microarray analyses using Genechip bovine genome arrays.

## Results

### Cell culture and hormone assays

**Figure 1:**
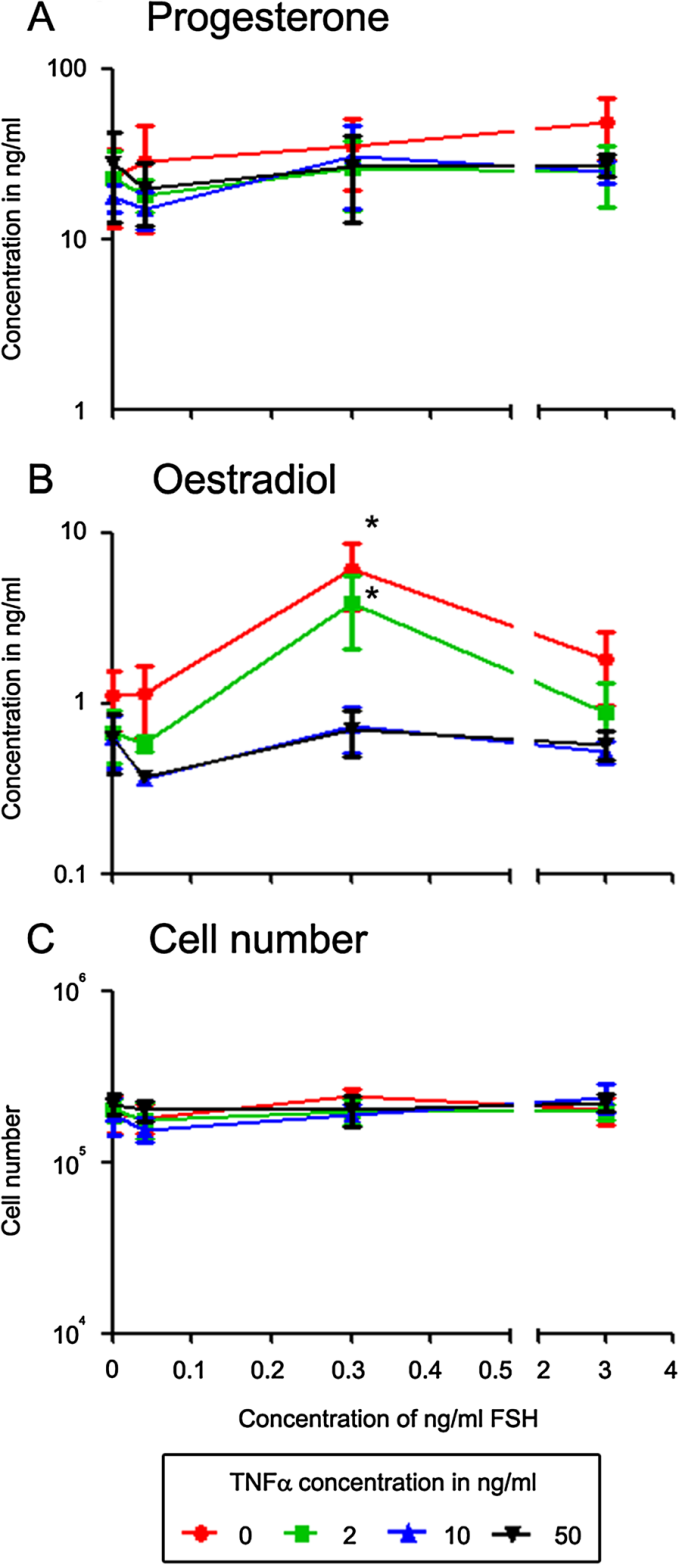
**Dose response effects of FSH and TNFα.** Oestradiol **(A)**, progesterone **(B)** and cell numbers **(C)** in cultured granulosa cells. The data represent the mean ± SEM (n = 4). Cell numbers represent viable cells per ml at the end of culture. The axes are logarithmically scaled. *indicates significantly different from controls (two-way ANOVA with Fisher’s LSD post hoc test, *P* < 0.05).

A dose response culture experiment was performed to determine the optimal concentrations of FSH and TNFα (Figure 1) to be used for the microarray experiments. On the basis of this experiment it was shown that oestradiol production was highest with a FSH dose of 0.33 ng/ml, being significantly different from values observed at all other FSH concentrations in the absence of TNFα. Ten ng/ml of TNFα was sufficient to reduce this effect to the control level. Progesterone levels and viable cell number at the end of the culture period were not significantly (*P* > 0.05) affected by either FSH or TNFα treatment. On the basis of these results, 0.33 ng/ml FSH and 10 ng/ml TNFα were selected for the culture of cells to be used for microarray analyses.

**Figure 2:**
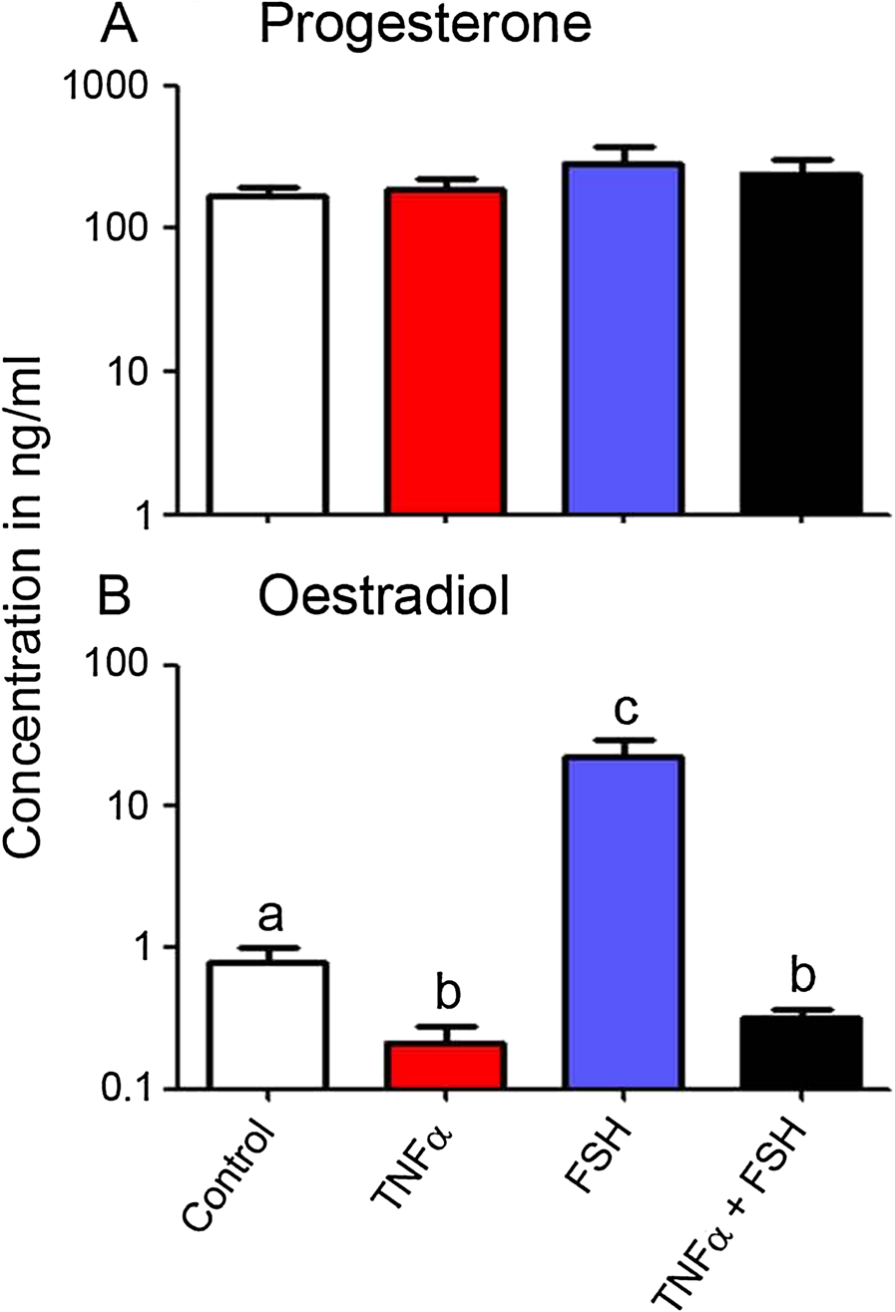
**Concentrations of hormones from granulosa cells cultured for microarray analyses.** Oestradiol **(A)** and progesterone **(B)** measured in media from cells cultured in the presence or absence of 0.33 ng/ml FSH and 10 ng/ml TNFα (n = 4 each). The axes are logarithmically scaled. Mean concentrations of oestradiol ± SEM in ng/ml for the different treatments were: 0.8 ± 0.2 (control), 0.2 ± 0.1 (TNFα), 22.4 ± 6.7 (FSH) and 0.3 ± 0.1 (TNFα + FSH). Corresponding values for progesterone were: 167 ± 29 (control), 187 ± 40 (TNFα), 289 ± 91 (FSH) and 243 ± 63 (TNFα + FSH). Significantly different values (two-way ANOVA with Fisher’s LSD post hoc test, *P* < 0.05) are indicated by different letters.

The results of the hormone secretion in the cultures of granulosa cells which were used for the microarray analyses are shown in Figure 2. FSH stimulation of the cultures produced a greater than 20-fold induction (*P* < 0.05) of oestradiol secretion by the granulosa cells compared with the control cells (Figure 2). Conversely, TNFα treatment caused a > 65% reduction in the amount of oestradiol secretion under basal conditions (*P* < 0.05) and completely abolished the FSH-induced rise in oestradiol secretion (*P* < 0.05). Progesterone levels averaged approximately 200 ng/ml across all cultures, trending higher for the FSH-treated culture, but no significant differences were observed between the treatments (Figure 2). This confirms that the cultured granulosa cells maintained a hormone production profile characteristic of non-luteinised cells, and responded to FSH and TNFα as anticipated.

### Hierarchical clustering and principal component analyses (PCA)

**Figure 3:**
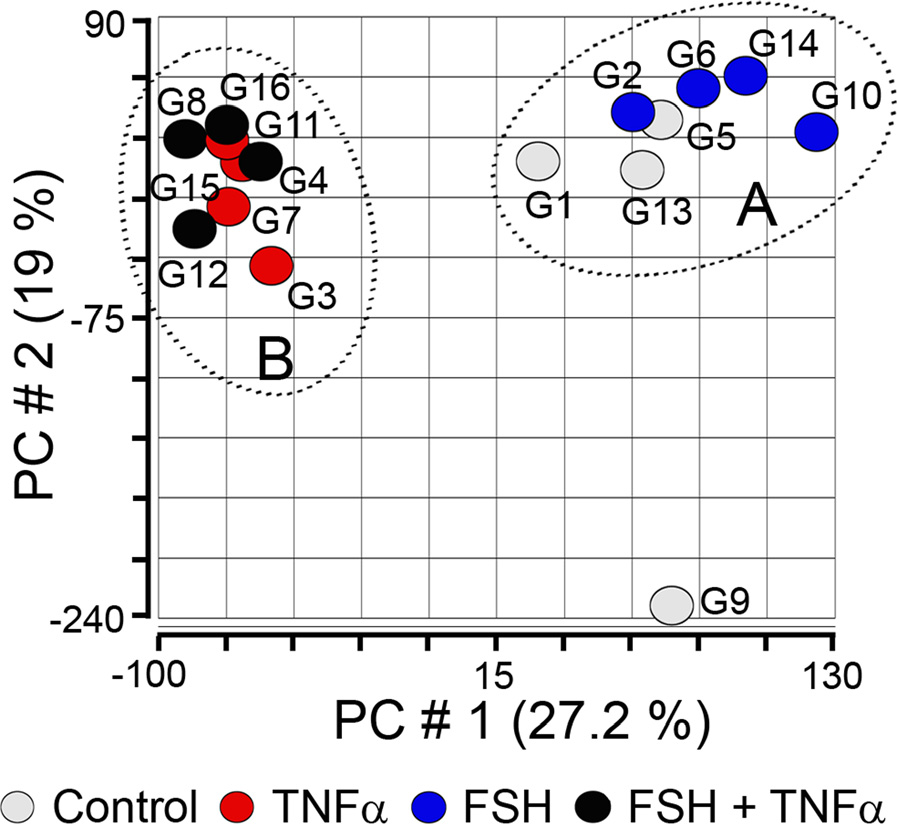
**Unsupervised PCA of the granulosa cell arrays using Partek.** The arrays are numbered (n = 4 per treatment) as follows: control granulosa (G1, G5, G9 and G13 in grey); FSH-treated (G2, G6, G10 and G14 in blue); TNFα-treated (G3, G7, G11 and G15 in red) and TNFα plus FSH-treated (G4, G8, G12, and G16 in black). The graph is a scatter plot of the values for the first (X) and second (Y) principal components based on the correlation matrix of the total normalised array intensity data. The groups of arrays **(A)** and **(B)**, encircled by the dotted lines, represent related arrays based on patterns of total gene expression determined from the PCA. The G9 array had a considerably different pattern of gene expression from all the others and was therefore considered to be a statistical outlier and excluded from further analysis.

These analyses were performed on the sixteen microarrays, four from each treatment group, as presented in Figure 3 (PCA) and Additional file 1: Figure S1 (hierarchical clustering). These figures show that culture sample G9 from the ‘control’ group was quite different in signal intensity across most probe sets on the chip from the other controls, and in fact all other arrays. This result prompted us to consider this array as a statistical outlier, and it was excluded from further analyses. The other arrays formed two clusters on the basis of distribution of signal intensity, the ‘TNFα and TNFα + FSH’ groups (B in Figure 3), and the ‘control and FSH’ groups (A in Figure 3). PCA was also conducted for the TNFα and the TNF + FSH-treated groups (n = 8), and then for the control and the FSH-treated groups (n = 7). In both cases, for each comparison no differences were observed between the FSH treated and the non FSH-treated cells (Additional file 2: Figure S2). ANOVA demonstrated that no genes were different by more than 2-fold with an FDR of *P* < 0.05. We can therefore assume that under our experimental culture conditions, FSH alone did not have a substantial effect on granulosa cell total gene expression, whereas TNFα had a major effect and actually overrode any effects of FSH when treated in combination.

### Quantitation of gene expression by RT-PCR

**Figure 4:**
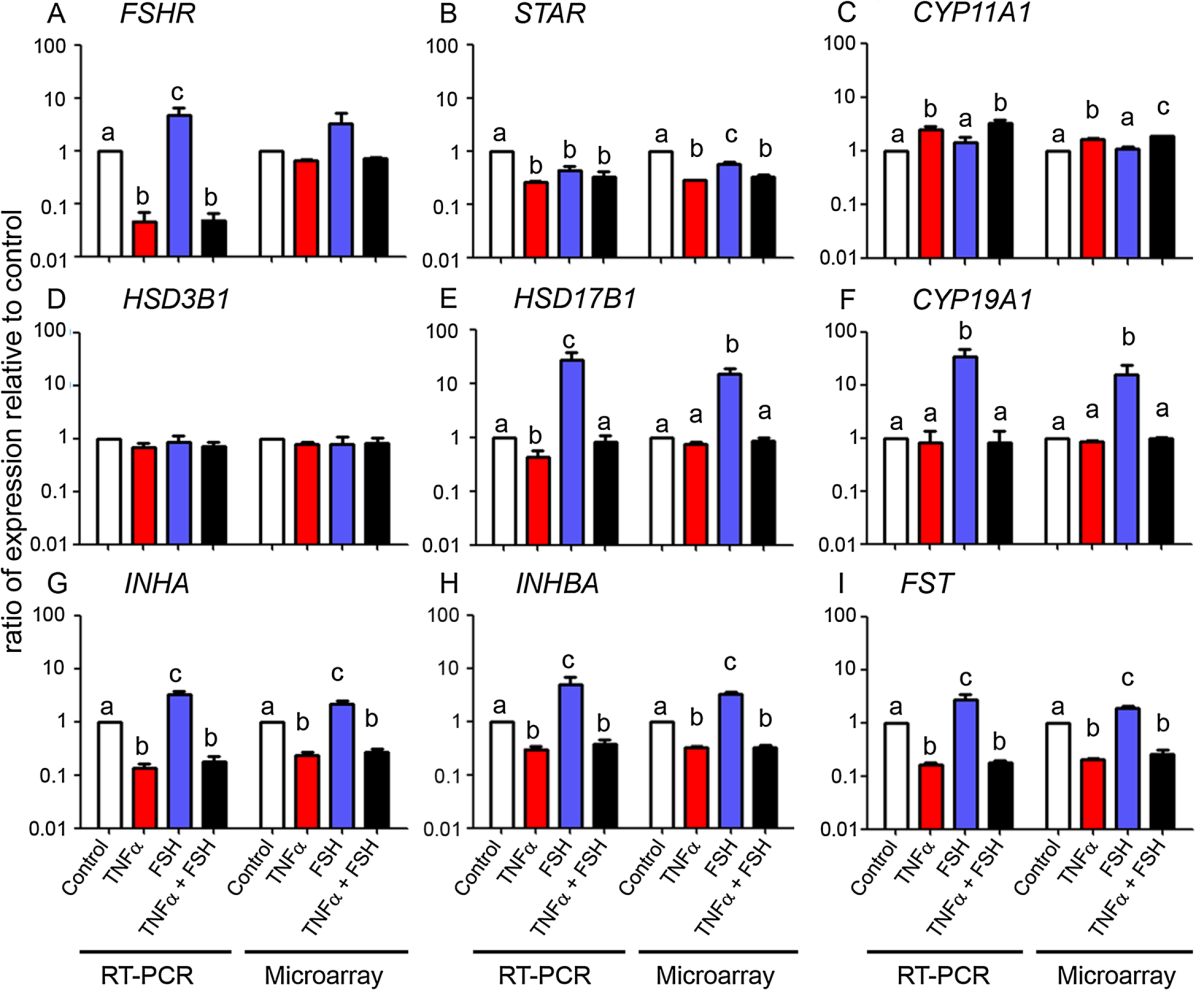
**Gene expression by qRT-PCR.** The data are shown on a log scale as the mean ± SEM (n = 4 each, except microarray control group n = 3) for the ratio of gene expression with treatments relative to control (set at 1.0). qRT-PCR values were determined from the ΔΔCt for the target genes **(A – I)**, relative to β-actin (*ACTB*), and the microarray values are signal intensities (normalised but not log transformed). Significantly different results for treatments used one-way ANOVA with Fisher’s LSD post hoc test; *P* < 0.05.

Figure 4 shows the quantitative RT-PCR results for some of the genes which were significantly affected by FSH and/or TNFα treatment of granulosa cells in culture: *FSHR* (A)*,* the steroidogenic genes *CYP19A1* (F)*, CYP11A1* (C)*, HSD3B1* (D), HSD17B1 (E) and *STAR* (B)*,* and other genes *INHA* (G)*, INHBA* (H) and *FST* (I). These genes are known to be active during antral follicular expansion when the granulosa cells are responsive to higher levels of FSH [22,23]. All genes except *STAR, CYP11A1* and *HSD3B1* were up regulated by FSH treatment alone. TNFα, whether alone or in combination with FSH, decreased expression of the following genes below the level of the control: *FSHR*, *STAR*, *INHA, INHB* and *FST* and the steroidogenic genes *HSD17B1* and *CYP19A1* to the level of the control. The decrease in *CYP19A1*, the gene encoding aromatase, the key enzyme of oestradiol synthesis, explains the observed decline in oestradiol production in the cultures under the influence of TNFα (Figure 2).

### Differential gene expression analyses

Comparison of all treatments against the control group by ANOVA with a three-fold change and a statistical cut-off of *P* < 0.05 produced a differentially-regulated list of 288 genes for the TNFα-treated arrays compared with the controls, and 232 genes were common to both TNFα datasets and regulated in the same direction and to approximately the same degree (Additional file 3: Figure S3 and Additional file 4: Table S1). These numbers reflect the results of the unsupervised statistical analyses.

Although FSH was shown by qRT-PCR and microarray analyses to statistically alter a number of specific genes (Figure 2), comparison of all treatments by ANOVA determined that no genes were differentially regulated by FSH treatment more than 2 fold with an FDR of *P* < 0.05. Additionally the hierarchical clustering and PCA analyses (Figure 3 and Additional file 1: Figure S1) did not indicate any major effects of FSH. We therefore compared TNFα ± FSH treated cultures (n = 8) with the control ± FSH treated cultures (n = 7) and generated lists of differentially expressed genes as shown in Table 1. Lists of genes which were three-fold differentially expressed between these conditions are presented in Tables 2 and 3 and the full annotations of these genes are presented in Additional file 5: Table S2. This list was imported into Ingenuity Pathway Analysis (IPA) and Gene Ontology Enrichment Analysis Software Tool Kit (GOEAST) for functional pathway and network analyses.

**Table 1:** The number of probe sets which were differentially regulated between control (± FSH) and TNFα (± FSH)-treated granulosa cell cultures

**Fold change**	**Up regulated**	**Down regulated**	**Total**
>2	869	463	1332
>3	400	127	527
>4	234	161	295
>6	107	12	119
>10	40	0	40

Numbers were determined by a one-way ANOVA with a FDR *P* < 0.05 for multiple comparisons using Partek.

**Table 2:** Genes which were >3-fold up regulated in TNFα (± FSH)-treated granulosa cells compared with control (± FSH) cells

**Gene symbol**	**Fold change**	**Gene symbol**	**Fold change**	**Gene symbol**	**Fold change**
	**(TNF**α **vs non TNF**α **treated)**		**(TNF**α **vs non TNF**α **treated)**		**(TNF**α **vs non TNF**α **treated)**
**Cell death**					
*TGM2*	31	*XAF1*	6.8	*IER3*	3.6
*CASP4*	15.2	*BCL2A1*	5.3	*PYCARD1*	3.3
*IFI6*	9.2	*SAMD9*	4.9	*TNFSF13B*	3.2
*BEX2*	8.6	*EGLN3*	4.5	*RIPK4*	3.1
*BIRC3*	8.6	*TRAF1*	4.3		
**Cell morphology**					
*KRT8*	10	*ERP27*	3.5	*NCK2*	3.1
*STMN2*	7.7	*PDPN*	3.5	*TACC1*	3.1
*MAP2*	4.7	*FMNL3*	3.4	*GPHN*	3.0
*DCLK1*	4.5	*MSN*	3.4	*MFAP2*	3.0
*KRT18*	4.2	*EMP3*	3.3		
**Cytokines, hormones and receptors**					
*GPR77*	61.5	*CD83*	6.3	*PLAUR*	3.8
*CD44*	21.5	*ROBO1*	6.1	*HLA-DQA1*	3.7
*IFI44*	14.9	*IFIT2*	6.0	*NTRK1*	3.6
*CD19*	14.2	*HLA-A*	5.9	*IL36A*	3.6
*IFI16*	13.6	*HLA-DQA2*	5.9	*EMR3*	3.5
*PTGER4*	13.2	*IL1RN*	5.9	*TCRA*	3.5
*CCL2*	13.1	*GPR68*	5.2	*TGFB1*	3.5
*SLCO4A1*	12.3	*HLA-DMB*	5.0	*GKN2*	3.4
*CD200*	10.8	*ICAM1*	4.7	*IFITM1*	3.4
*CXCL10*	10.8	*HLA-DRA*	4.6	*HLA-DRB3*	3.3
*CD40*	10.1	*LYVE1*	4.6	*FGF2*	3.2
*IFI27*	9.5	*IL1R1*	4.5	*VIPR2*	3.2
*PLXND1*	9.4	*NR4A2*	4.3	*GPR56*	3.1
*IFIH1*	7.1	*OLR1*	4.0	*CD72*	3.1
*JAG1*	6.7	*NRG1*	3.9	*IGF2*	3.1
*CCL5*	6.4	*PDGFB*	3.8		
**Extracellular matrix and synthesis**					
*TNC*	52.3	*COL15A1*	9.4	*FMOD*	4.0
*COL6A1*	14.5	*COL16A1*	5.0	*AGRN*	3.8
*LOXL4*	8.8	*COL5A3*	4.6	*FN1*	3.3
**Intercellular and cell to matrix adhesion**					
*BST2*	6.4	*THBS3*	3.6	*CEACAM8*	3.2
*VCAM1*	5.3	*SPP1*	3.6	*CDH1*	3.1
*CD82*	4.8	*CD9*	3.6		
*ITGA5*	4.5	*NUAK2*	3.4		
**Ion transport**					
*KCNMA1*	8.9	*FXYD3*	3.9	*CLCA3P*	3.1
*CACNA1G*	6.9	*GRIA3*	3.7	*SLC25A12*	3.0
*CNGA3*	4.3	*TPCN1*	3.4		
**Proteolysis and inhibition**					
*SLPI*	58.6	*MMP2*	5.0	*CTSH*	3.5
*UBD*	17.1	*PSMB10*	3.9	*TFPI2*	3.5
*SERPINB2*	14.9	*PSMB8*	3.8	*TRIM21*	3.2
*SERPINA5*	12.5	*ADAMTSL4*	3.8	*UBA7*	3.2
*ISG15*	10.8	*ERAP2*	3.8	*PRSS2*	3.1
*ADAM23*	8.3	*USP18*	3.7	*CFD*	3.1
*A2M*	5.6	*TIMP3*	3.7	*CSTB*	3.1
*PSMB9*	5.1	*C1S*	3.6		
**Transcriptional regulation**					
*GFI1*	14.1	*IRF1*	4.7	*NFKB2*	3.7
*NFKBIA*	9.8	*HEYL*	4.7	*NFKBIZ*	3.6
*FOXA3*	9.0	*HES4*	4.6	*H19*	3.5
*MYB*	8.4	*IRF8*	4.2	*SNAI1*	3.4
*MIR147*	5.3	*LRRFIP1*	4.0	*NOSTRIN*	3.4
*TBX3*	5.2	*NUPR1*	4.0	*DTX1*	3.3
*BHLHE40*	4.8	*RBPMS*	3.9	*FOXS1*	3.3
*TCF7*	4.8	*IKZF3*	3.8	*STAT5A*	3.1
**Transport**					
*RTP4*	14.5	*RAB3IP*	3.8	*SLC37A1*	3.5
*ABCC3*	8.0	*ANKH*	3.7	*SLCO2A1*	3.4
*TF*	4.1	*TAP1*	3.5	*SYNGR1*	3.1
**Other enzymes**					
*PRKCB*	19.3	*PNPO*	5.0	*TPST2*	3.9
*PTGIS*	12.2	*PIK3CD*	5.0	*PRKCQ*	3.9
*ALDH1A3*	11.7	*GBP4*	4.8	*CA5B*	3.8
*OAS2*	10.5	*MIOX*	4.7	*PARP14*	3.7
*PON3*	9.3	*ADCY2*	4.7	*PARP12*	3.7
*DDC*	8.7	*ADH6*	4.5	*LRAT*	3.5
*IDO1*	7.7	*UMPS*	4.4	*CKB*	3.4
*MX2*	7.5	*CA2*	4.4	*CAMK1D*	3.3
*HSD11B1*	7.0	*RSAD2*	4.3	*FBP1*	3.3
*MX1*	6.6	*PPP2R3C*	4.3	*PARP9*	3.2
*OAS1*	5.5	*BCAT2*	4.3	*PNKD*	3.2
*ABHD3*	5.2	*SQRDL*	4.1	*CA8*	3.1
*GLDC*	5.2	*ISG20*	4.0		
*XDH*	5.2	*APOBEC3B*	3.9		
**Other signalling**					
*ENTPD3*	17.7	*DNER*	5.8	*ARHGAP24*	4.8
*ARHGEF5*	13.6	*RASAL1*	5.2	*DDX58*	3.3
*NFKB1A*	9.8	*TNIP1*	5.5	*WNT11*	3.2
*ARHGAP29*	8.7	*ARHGEF11*	5.1	*NKG7*	3.4
*ANXA8L1*	8.0	*ARRDC2*	4.3	*CDC42EP1*	3.3
*SLAMF8*	7.4	*RGS16*	4.1	*PDE4B*	3.5
*TAC3*	8.7	*SHISA3*	4.1	*IFI30*	3.2
*GAL*	4.6	*GBP5*	4.0	*RNF128*	3.0
*DAPP1*	5.0	*ARHGDIB*	3.8		
**Other**					
*TREM1*	11.2	*TMEM45B*	4.6	*DPY19L1*	3.6
*PLAC8*	7.7	*MS4A8B*	4.6	*NELL2*	3.6
*PARM1*	6.5	*SAA3*	4.6	*TMEM140*	3.4
*EPB41L3*	6.3	*SELENBP1*	4.4	*CCDC85C*	3.4
*FATE1*	6.2	*CRYBB1*	4.4	*OAF*	3.2
*TRIM47*	5.4	*HPCAL1*	4.2	*TMED6*	3.2
*SPARCL1*	5.4	*UNC13D*	4.0	*ABTB2*	3.2
*NUCB2*	5.2	*EPSTI1*	3.9	*CRIP2*	3.2
*PDLIM4*	4.8	*HSPB6*	3.8	*CSRP2*	3.1

Genes are categorised by function and listed in descending order of fold change in each category. The association of genes with functional terms in this table was determined manually for each gene based on information obtained through the Entrez Gene database of NCBI. The level of fold change and statistical significance ( ≥ 3-fold change, *P* < 0.05) were determined using the step up Benjamini-Hochberg post-hoc test for multiple corrections following one way ANOVA.

**Table 3:** **Genes which are >3-fold down regulated in TNF**α **(± FSH)-treated granulosa cells compared with control (± FSH) cells**

**Gene symbol**	**Fold change**	**Gene symbol**	**Fold change**	**Gene symbol**	**Fold change**
	**(TNF**α **vs non TNF**α **treated)**		**(TNF**α **vs non TNF**α **treated)**		**(TNF**α **vs non TNF**α **treated)**
**Apoptosis**					
*TRIB2*	5.7				
**Cell morphology**					
*DACT1*	4.1	*ACTG2*	3.3	*MYO1B*	3.1
**Cytokines, hormones and receptors**					
*ROBO2*	7.9	*HEG1*	5	*PTN*	3.5
***EFNA5***	7.3	*PLXNC1*	4.4	*AMH*	3.4
*FST*	6.2	*PPARG*	4.2	*NR5A2*	3.1
*INHA*	5.8	*ANGPT2*	3.8	*KIT*	3
*INHBA*	5.8	*CD36*	3.6		
*NMB*	5.5	*EPHA7*	3.6		
**Extracellular matrix and synthesis**					
*SPOCK2*	5.0	*FBLN5*	4.8	*EFEMP1*	3.3
*SRGN*	5.5				
**Intercellular and cell to matrix adhesion**					
*CYR61*	5	*CAV1*	4.1	*CTNNAL1*	3.3
*TSPAN5*	4.9	*TNFAIP6*	3.5	*CLDND1*	3.3
*NPNT*	4.6	*SMOC2*	3.4		
**Ion transport**					
*KCNN2*	3.6	*ATP4B*	3.1		
**Proteolysis and inhibition**					
*TLL2*	4.8	*PRSS35*	3.4	*SERPINE2*	3.0
**Transcription regulation**					
*TOX*	4.1				
**Transport**					
*MAL2*	4.0	*SLC2A3*	3.7	*NPC1*	3.0
**Other enzymes**					
*CHST8*	8.4	*DDAH1*	4.4	*PLA2G7*	3.5
*VNN1*	6.4	*HPSE*	4.1	*GCLC*	3.4
*STC1*	5.8	*MVK*	3.7	*TRIM2*	3.2
*PPAP2B*	5.5	*PDK4*	3.6		
*HSD17B1*	4.8	*GYLTL1B*	3.6		
*PTGS2*	4.6				
**Other signalling**					
*TPD52L1*	7.3	*DAB2*	3.4	*CCND2*	3.1
*RGS2*	4.6	*FGD5*	3.2	*SMAD2*	3.1
*SORBS2*	4.4	*RASL11B*	3.2		
*RGMB*	3.8	*NTS*	3.2		
**Other**					
*ZNF608*	4.6	*EEPD1*	4.4	*CAPRIN2*	3.5
*SUSD4*	4.5	*LINGO2*	3.7	*LGALS3*	3.1

The level of fold change and statistical significance ( ≥ 3-fold, *P* < 0.05) was determined by one-way ANOVA with post-hoc Benjamini-Hochberg testing for multiple corrections. The association of genes with functional terms in this table was determined manually for each gene based on information obtained through the Entrez Gene database of NCBI. Genes are listed in descending order of fold change in each category.

### IPA and GO enrichment analysis of TNFα regulated dataset

**Figure 5:**
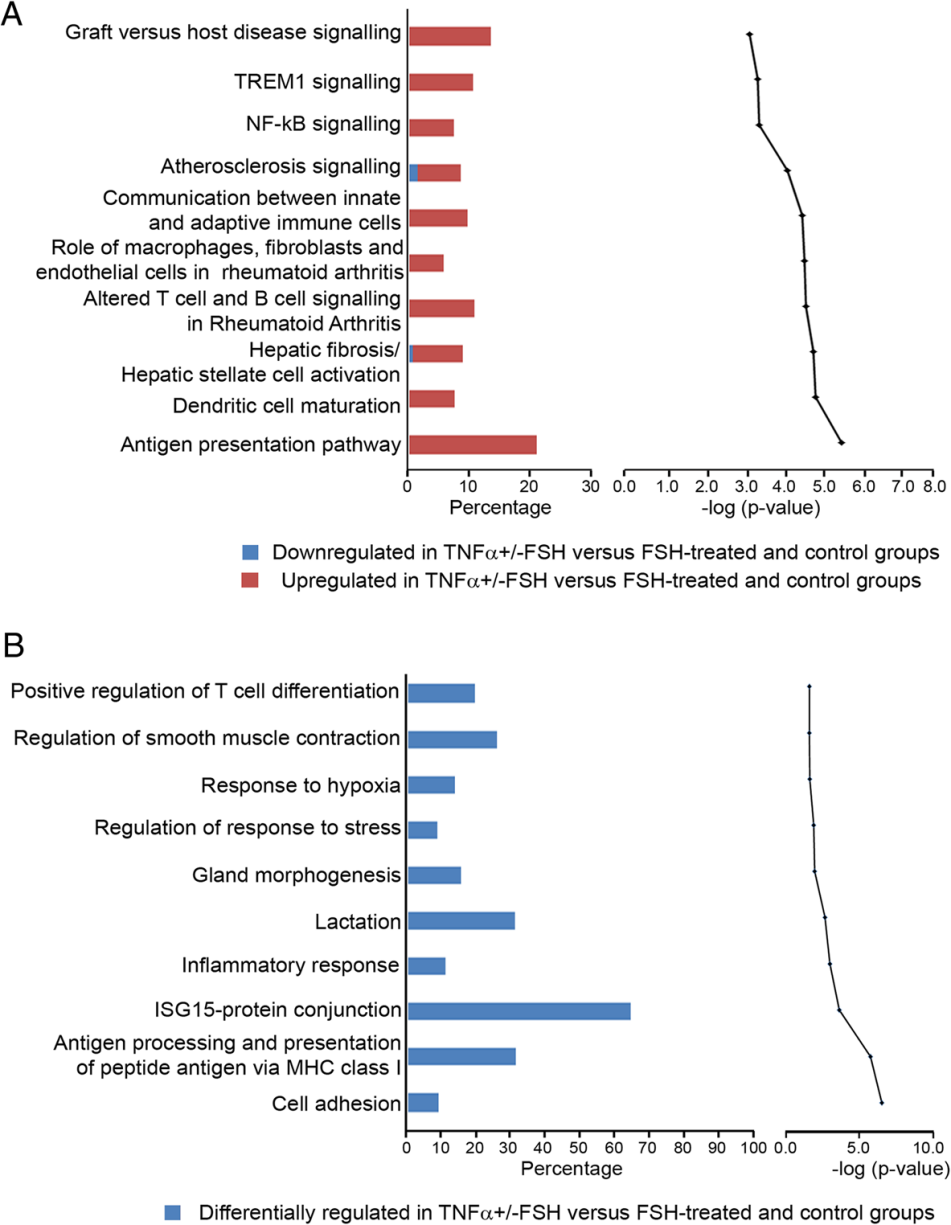
**Top ten canonical pathways and highly enriched GO terms.** Determined for genes differentially regulated between control (± FSH) and TNFα (± FSH)-treated granulosa cells (n = 527 probe sets, 379 genes mapped to IPA). In **(A)** the bar charts represent the percentage of genes from the dataset that map to each canonical pathway in IPA (up regulated in red and down regulated in blue for TNFα (± FSH)-treated granulosa cells). The line chart ranks the pathways derived from the highest to lowest degree (bottom to top) of association based on the value of a Benjamini-Hochberg test for multiple corrections. In **(B)** the bar charts represent the percentage of differentially-regulated genes between control (± FSH) and TNFα (± FSH)-treated granulosa cells. Only the most specific subcategories of GO terms under biological process which were considered significant by the Benjamini-Yuketeli test (*P* < 0.05) were ranked.

Genes from the dataset that were differentially regulated > 3 fold and *P* < 0.05 between TNFα ± FSH and control ± FSH were mapped to canonical pathways in IPA and shown in Figure 5A. Many of the pathways were associated with immune cell function and inflammatory response pathways. Several of these pathways contain genes such as the nuclear factor-kappa- B complex (NF-κB) genes, *NFKB1A* and *NFKB2,* interleukin receptor 1 and the cell survival gene *PIK3R1.* The GO enrichment analysis for this dataset (Figure 5B), also showed over-representation of processes such as antigen presentation, inflammation and stress response.

We also used IPA upstream regulator analysis to identify upstream transcriptional regulators. Upstream regulators were predicted using a Fisher’s exact *t*-test to determine the probability that genes from the dataset correspond with targets which are known to be activated or inhibited by those molecules based on current knowledge in the Ingenuity database. Table 4 reveals a number of upstream regulators which were predicted to be influenced by TNFα treatment. It contained the inflammatory response mediators *IL1B* and *IF1B*, and the matrix encoding gene *FN1.* Interestingly, the interleukin receptor antagonist *IL1RN* was predicted to be down regulated, but it was shown to be up regulated according to the array intensity data.

**Table 4:** **Upstream regulators determined by IPA to be activated or inhibited by TNF**α **(± FSH) treatment of cultured granulosa cells**^†^

**Gene symbol or molecule**	**Full gene name**	**Actual fold change**	**Activation z-score**	***P *** **value of overlap**
TNF	Tumour necrosis factor		6.52	7.03E-39
IFNG	Interferon gamma		6.75	6.34E-35
IL1B	Interleukin 1, beta		4.76	9.88E-28
Tretinoin	All-trans retinoic acid		4.12	7.15E-25
IFNB1	Interferon beta 1		3.66	8.03E-20
OSM	Oncostatin M		4.29	5.93E-16
CD40	CD40 molecule, TNF receptor superfamily member 5	10.1	3.25	2.36E-13
TGM2	Transglutaminase 2	31.0	3.34	1.05E-12
FN1	Fibronectin 1	3.3	3.05	7.85E-11
ESR1	Estrogen Receptor 1		3.07	1.99E-07
HTT	Huntingtin		3.32	8.34E-07
IL1RN	Interleukin 1 receptor antagonist	5.9	−3.60	8.11E-19

^†^Genes >3-fold differentially regulated between TNFα (± FSH) and control (± FSH) were used for this analysis.

The *P* value of overlap is the calculated statistical significance of overlap between genes from the dataset and genes that are known to be regulated by the upstream regulator using Fisher’s exact test.

The bias-corrected z-score is used to infer the activation states of transcriptional regulators. It is calculated from the proportions of genes which are differentially regulated in an expected direction based on the known interactions between the regulator and the genes present in the Ingenuity database. Those genes with a z-score greater or less than two are considered to be either activated or inhibited respectively.

**Figure 6:**
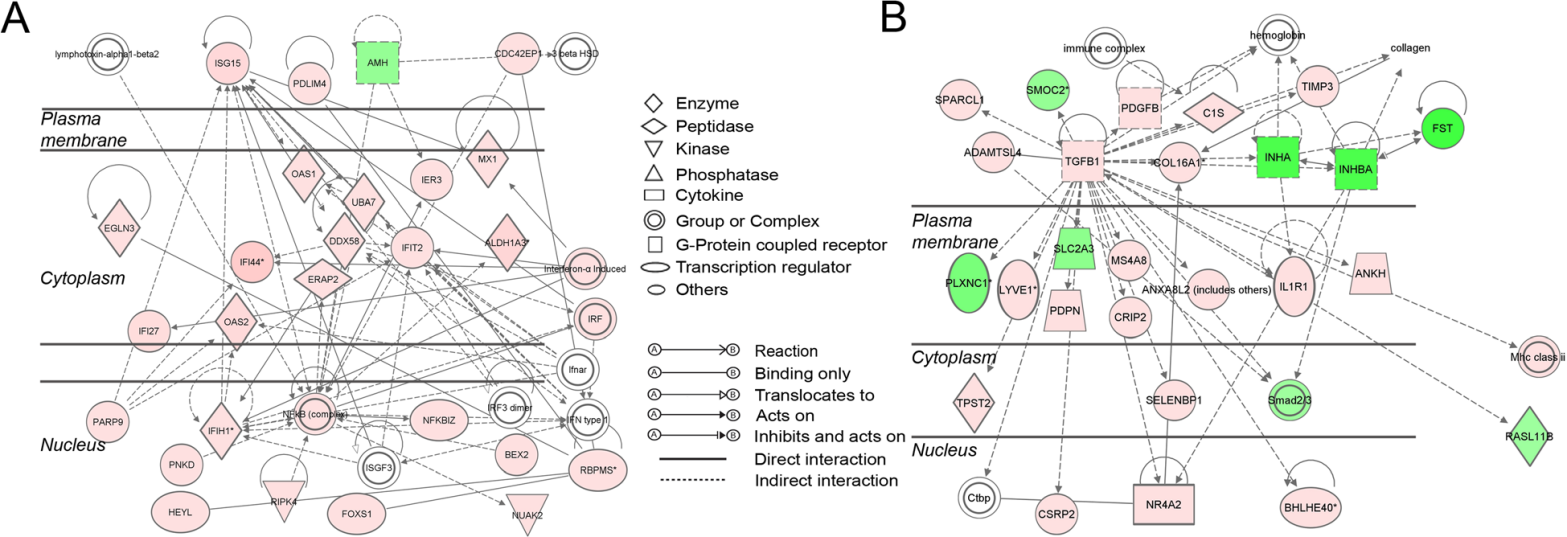
**The top two networks (A and B) determined in IPA.** Genes were differentially regulated between TNFα (± FSH)-treated and control (± FSH)-treated granulosa cells (n = 379 genes mapped to IPA). The networks were generated by a modified triangle connectivity algorithm based on the proportion of known interactions between molecules in this dataset (focus molecules) and others in the IPA database. Both networks were scored at 44 (−log of *P* value of Fisher’s exact *t*-test), and mapped 26 genes each from the differentially expressed dataset. Interactions between molecules are shown as explained in the legend, with focus molecule symbols highlighted in colour, based on up (red) or down (green) regulation and increasing colour intensity with degree of fold change.

The two highest scoring gene networks generated in IPA from our dataset for the effect of TNFα on the cultured granulosa are displayed in Figure 6. Network A shows an emphasis on innate immune response genes including several which are induced by interferon such as *IFIT2*, *IFI44, IFIH1* and *IFI27*, which are all up regulated. Other genes in this network have some involvement with apoptotic signalling, namely *RIPK4, FOXS1* and *BEX2*. The molecule(s) forming most interactions in this network is the NF-κB complex, located within the nucleus. The other network (B) shows a focus around *TGFB1* with interactions between other genes such as *PDGF*, *COL16A1* and *ADAMTSL4*. There is also connectivity with the down-regulated genes *INHA, INHBA* and *FST*, which are all known to play a role in folliculogenesis as previously mentioned. Other genes which were highly activated due to TNFα treatment (Table 2 and Additional file 4: Table S1), included *GPR77* and *SLP1* (55-fold up regulated), *PRKCB* (20-fold), *COL6A1* (13-fold), *KRT8* (10-fold) and *HSD11B1* (6-fold). Amongst the down-regulated group of genes, *CHST8* (8-fold) (Table 3 and Additional file 4: Table S1) may have a novel intra-follicular role.

## Discussion

This study examined the effects of the treatment with FSH and TNFα, separately or combined, on granulosa cells cultured under serum-free conditions and maintained in a non-luteinised state. The effect of FSH alone at 0.33 ng/ml paralleled the result of a previous study by Glister et al*.*[17], with similar increases in oestradiol production and expression of follistatin and inhibin A confirming the robustness of this physiologically relevant *in vitro* model used in the current experiments. Moreover, our qRT-PCR findings confirmed the ability of FSH to up regulate expression of its cognate receptor (FSHR) as reported previously [23,24] although a statistically significant difference was not detected by microarray analysis. With the exception of FSHR, there was excellent agreement between microarray and qRT-PCR data with respect to treatment effects on the other eight transcripts used for validation purposes. The ability of TNFα to suppress the production of oestradiol in our experiment was also expected from results of previous studies in serum-free [13] and serum-supplemented [15] culture systems.

The unsupervised array analyses and the numbers of genes differentially regulated, show surprisingly perhaps, that in our experiment FSH alone had a minor effect on total gene expression, compared with TNFα, where many genes were differentially regulated. The effects of FSH were limited to stimulation of energy metabolism and steroidogenesis in overall terms, in comparison with TNFα which mainly influenced inflammatory pathways and molecules. Clearly the main specific effect of FSH treatment was to stimulate oestradiol production by up regulation of aromatase expression (*CYP19A1*). The production of oestradiol and the concomitant activation of the folliculogenesis regulating genes for inhibin A and follistatin occur through recognised FSH cascade signalling involving cyclic AMP and protein kinase A [24]. The relatively low dose of FSH (0.33 ng/ml) used to treat the cells in our microarray experiment was selected as being optimal for promoting oestradiol secretion and was insufficient to induce an increase in progesterone synthesis or proliferation of the granulosa cells in culture. However, there was transcriptional activation of the cyclin B1 and B2 genes, which indicates an increase in mitotic activity, although a net increase in cell number was not observed under the culture conditions used. The endocrine functions of granulosa cells appeared to be down regulated upon TNFα treatments with reductions in *FST* (6.2 fold), *INHA* (5.8 fold), *INBA* (5.8 fold), *AMH* (3.4 fold).

*CHST8* was also down regulated in the TNFα-responsive datasets. The encoded enzyme is a sulphur transferase that sulphates *N*-acetylgalactosamine β1,4 linked with *N*-acetylglucosamine (LacdiNAc) moieties on certain glycoproteins prior to secretion [25]. Sulphation of these structures can modulate the activity of these molecules by affecting the kinetics of binding [26] and increases their rate of clearance from the body [27]. Glycodelin, an immune mediator, is produced by the granulosa cells at antral stages and possesses these LacdiNAc moieties. Glycodelin is taken up by the cumulus cells, where it is deglycosylated, loses immunosuppressive function and acquires properties beneficial to the fertilisation process [28]. It may be possible that sulphation may also play a role in determining the activity of glycodelin, but confirmation of this would require further investigation.

It is known that TNFα exerts its apoptotic effects through the Type I receptor (TNFR1), whereas other pro-inflammatory actions on growth and differentiation are mediated via the Type II receptor (TNFRII) as previously reviewed by Matsuda *et al.*[16]. Since we did not observe any effect on viable cell number after 4 days exposure to TNFα, this might suggest that TNFα did not induce apoptosis in the granulosa cells and may act predominantly via TNFRII in our culture system to activate a pro-inflammatory cascade that modifies other aspects of cell function including matrix remodelling and up regulation of antigen presentation molecules. Many of the genes whose expression in granulosa cells was up regulated by TNFα are often associated with innate immune responses. This reinforces recent evidence that granulosa cells can act as immune sensors and play an active role in initiating protective inflammatory responses to bacterial pathogens, recognised via interaction of pathogen-associated molecular patterns (PAMPs) such as lipopolysaccharide, with toll-like receptor 4 (TLR4) on the cell surface [21,29,30]. Indeed, bovine granulosa cells were recently shown to express a full complement of TLRs. Moreover, functional inflammatory responses to PAMPs interacting with TLR2 and TLR4 were demonstrated [21].

The genes influenced by TNFα treatment were generally associated with endocrine function, apoptosis, inflammation, and degradation as were expected from previous studies. In our culture system, TNFα alone did not cause any net loss of cells. Some pro-apoptotic (*XAF1, CASP4*), but more anti-apoptotic genes (*TGM2, BCL2A1, BIRC3, TNIP1*), in fact, appeared to be stimulated by TNFα. It is likely that the genes which act to block the apoptotic process are responding as a cellular survival mechanism, although some may be directly activated via the TNFα signalling pathway. The blocking effect of TNFα on FSH-induced oestradiol production has been shown previously to be suppressed by treatment with peroxisome proliferator-activated receptor (PPAR) γ ligands [31], and in this study *PPARG* expression was inhibited by TNFα, indicating that this lipid metabolism pathway was also involved. *HSD11B1* encodes a key enzyme in glucocorticoid metabolism and has been previously shown to be activated by TNFα via the regulatory gene *CEBPB* in cultured cells [32].

The genes identified as being most highly up regulated by TNFα include *TGM2* (31-fold), *GPR77* (62-fold), *SLPI* (59-fold) and *TNC* (52-fold), none of which have previously been noted in granulosa cells. *TGM2* catalyses the cross-linking of proteins and the conjugation of polyamines to proteins. It is also implicated as a positive regulator of the inflammatory response, NF-κB signalling and cell adhesion [33]. *GPR77* (62-fold) is one of several receptors for the C5a molecule, a major chemotactic and pro-inflammatory product of the complement cascade activated during the innate immune response [34]. *SLPI* (59-fold) was first characterised as a protease inhibitor but is now recognised as having additional properties including antimicrobial and immunomodulatory activities associated with the innate immune response. SLPI is up regulated by pro-inflammatory mediators and appears to have a tissue protective role [35,36]. *TNC* (52-fold) is an extracellular matrix molecule that is highly expressed during embryonic development but is normally present in low amounts in adult tissues. However, *TNC* expression is up regulated in pathological situations involving tissue injury, wound healing, inflammation and cancer. *TNC* influences cell migration, proliferation and cell signalling pathways through a variety of mechanisms including induction of pro-inflammatory cytokines [37].

## Conclusions

*In vitro*, the transcriptome of granulosa cells responded minimally to FSH compared with the response to TNFα. The response to TNFα indicated a reduction in endocrine function and an active process akin to tissue repair and remodelling as would occur upon atresia. Additionally there was an inflammatory response to TNFα that displays many features normally associated with immune cells.

## Methods

### Bovine ovaries and primary culture of granulosa cells

Bovine granulosa cells were isolated from adult bovine ovaries obtained from a local abattoir as described previously [17,18,38]. Contamination with theca cells was judged to be < 1% based on comparison of the relative expression of *CYP17A1* and *LHCGR* in freshly isolated granulosa cells and theca cells as determined by qRT-PCR (data not shown). For each experiment cells were pooled from approximately 50 individual 4–6 mm follicles and seeded at 5×10^5^ viable cells/ml into 24-well (microarray) or 75,000 cells/0.2 ml into 96 well plates (dose response) with four replicate wells per treatment. Cells were cultured for six days under defined serum-free conditions. The culture medium used consisted of McCoy’s 5A modified medium supplemented with 1% (v/v) antibiotic-antimycotic solution, 10 ng/ml bovine insulin, 2 mM L-glutamine, 10 mM HEPES, 5 μg/ml apo-transferrin, 5 ng/ml sodium selenite and 0.1% (w/v) BSA (all purchased from Sigma UK Ltd, Poole, Dorset, UK). The culture medium was supplemented with 10^-7^ mol/l androstenedione (Sigma UK Ltd, Poole, Dorset, UK) as a substrate for cytochrome P450 aromatase. Media were removed after 48 h and 96 h and replaced with fresh media containing treatments described below. Conditioned media were retained for hormone assays, and at the end of culture either viable cell number was determined (dose–response experiment) by neutral red uptake assay [17] or cell lysates were prepared (microarray experiment) using the lysis buffer component of the RiboPure RNA isolation kit (Ambion/ Life Technologies Ltd., Paisley, UK). Pooled lysates from replicate wells were stored at -80C until total RNA isolation. Each experiment was repeated four times using cells harvested from independent batches of ovaries.

### Treatments

Highly purified ovine FSH (NIADDK oFSH-19SIAPP) was supplied by NHPP, Torrance, CA, USA. Recombinant human TNFα was purchased from Sigma Aldrich, St Louis, MO, USA (Cat# T6674 with a stated endotoxin level <1 ng/ug). Treatments were dissolved in Hank’s balanced-salt solution containing 0.1% (w/v) BSA and stock solutions sterilized using 0.2 μm membrane filters before dilution in the culture medium. These treatments were applied on days 3 to 6 of culture for both the microarray and dose response experiments under the conditions specified above.

### Steroid immunoassays

The concentrations of oestradiol in conditioned media were determined by radioimmunoassay [39]. The detection limit of the assay was 2 pg/ml and mean intra- and inter-assay CVs were 6% and 9% respectively. Concentrations of progesterone in conditioned media were determined by competitive ELISA [40]. The detection limit was 0.1 ng/ml and mean intra- and inter-assay CVs were 8% and 11% respectively.

### Total RNA isolation, microarray analysis and quantitative RT-PCR

Total RNA was isolated from cultured cells for microarray analysis and for validation using qRT-PCR. RNA was isolated using the RiboPure™ RNA isolation kit (Ambion) according to the manufacturer’s instructions. RNA yield and quality were evaluated by spectrophotometry at 260/280 nm and agarose gel electrophoresis before submitting samples (n = 16) to an accredited Affymetrix service provider (Almac Diagnostics Ltd, Craigavon, Northern Ireland) for microarray analysis. For qRT-PCR analysis, first strand cDNA was synthesized from 1 μg of total RNA using the Reverse-iT™ reverse transcription kit (used according to manufacturer’s protocol; Abgene, Epsom, Surrey, UK) in a 20 μl reaction primed with random hexamers. Primers (see Table 5) were designed to amplify target sequences using Primer Express software (Applied Biosystems/ Life Technologies Ltd) or the online Primer Design Tool (NCBI/Primer-BLAST). In primer validation experiments, dissociation curve analysis and agarose gel electrophoresis were used to verify that each primer pair generated a single product of the predicted size. cDNA template log-dilution curves were used to demonstrate satisfactory PCR efficiency (> 85%) and linearity. PCR assays were carried out in a volume of 14 μl, comprising 5 μl cDNA template (1/50 dilution), 1 μl each forward and reverse primers (final concentration 0.4 μM) and 7 μl QuantiTect SYBR Green qPCR 2x Master Mix (Qiagen Ltd., Crawley, west Sussex, UK). Samples were processed on a StepOne^TM^ Plus real-time PCR instrument (Applied Biosystems/ Life Technologies Ltd.) with the following thermal cycling conditions: 15 min at 95°C (one cycle) followed by 15 s at 95°C and 1 min at 60°C (40 cycles).

**Table 5:** List of primers used for qRT-PCR

**Target**	**Genbank accession number**	**Forward primer 5′ to 3′**	**Reverse primer 5′ to 3′**	**Amplicon size (bp)**
*FSHR*	NM_174061	GCCAGCCTCACCTACCCCAGC	AATTGGATGAAGGTCAGAGGTTTGCC	75
*STAR*	NM_174189	TTTTTTCCTGGGTCCTGACAGCGTC	ACAACCTGATCCTTGGGTTCTGCACC	103
*CYP11A1*	NM_176644	CAGTGTCCCTCTGCTCAACGTCC	TTATTGAAAATTGTGTCCCATGCGG	99
*HSD3B1*	NM_174343	GCCACCTAGTGACTCTTTCCAACAGCG	TGGTTTTCTGCTTGGCTTCCTCCC	111
*HSD17B1*	NM_001102365	CGCATATTGGTGACCGGGAGCATA	AATCGCCAGACTCTCGCACAAACC	108
*CYP19A1*	NM_174305	CGCCACTGAGTTGATTTTTGCTGAGA	TAAGGCTTTGCGCATGACCAGGTC	301
*ACTB*	NM_173979	ATCACCATCGGCAATGAGCGGTTC	CGGATGTCGACGTCACACTTCATGA	128
*INHBA*	NM_174363	GAAGAGACCCGATGTCACCCAGC	TGTCGTCCTCTATCTCCACGTACCCG	113
*INHA*	NM_174094	GAGCCCGAGGACCAAGATGTCTCC	CCTCAGCCTCTCCAGCATCTGGC	91
*FST*	NM_175801	TGAGCAAGGAGGAGTGTTGCAGCA	CATCTGGCCTTGAGGAGTGCACATTC	301

For qRT-PCR analyses, the ∆∆Ct method [41] was used for comparison of the relative abundance of each mRNA transcript. Ct values for each transcript in a given sample were first normalised to the β-actin Ct value (which was uniform across all experimental groups: ANOVA *P* > 0.1). Resultant ∆Ct values for individual replicates within each treatment group were then normalised to the average ∆Ct value of the respective vehicle-treated control group. These ∆∆Ct values were finally converted to fold differences using the formula: fold difference = 2^(−∆∆Ct).^

### Statistical analyses

Results for hormone secretion (during final 96–144 h period of culture) were analysed using two-way ANOVA and are presented as means ± SEM based on four independent culture experiments. To reduce heterogeneity of variance, hormone data were log-transformed prior to statistical analysis. qRT-PCR data (from n = 4 independent granulosa cell batches) were statistically analysed (ANOVA and post-hoc Fisher’s LSD test) as ∆Ct values before conversion to fold-difference values for graphical presentation.

### Microarray

Following confirmation of the quality of the RNA and cDNA synthesis, hybridisations to GeneChip® Bovine Genome Arrays (Affymetrix, CA, USA) and scanning were performed according to Affymetrix protocols at the Almac Diagnostics Facility. All samples were analysed together as one lot using the same batch of arrays. First-strand cDNA synthesis was performed on two micrograms of RNA using a T7-linked oligo-dT primer, followed by second strand synthesis. *In vitro* transcription reactions were performed in batches to generate biotinylated cRNA targets, which were subsequently chemically fragmented at 95°C for 35 min. Ten μg of the fragmented, biotinylated cRNA was hybridized at 45°C for 16 h to Affymetrix GeneChip Bovine Genome Arrays, which contain 24,128 probe sets representing over 23,000 transcripts and variants, including 19,000 UniGene clusters. The arrays were then washed and stained with streptavidin-phycoerythrin (final concentration 10 μg/ml). Signal amplification was achieved by using a biotinylated anti-streptavidin antibody. The array was then scanned according to the manufacturer’s instructions. The scanned images were inspected for the presence of any defect (artefact or scratch) on the array.

### Treatment and analysis of microarray data

Non-biological signal variation due to possible array differences or hybridisation treatments were minimised by normalisation of the raw data using the Robust Multi-array Average (RMA) method [42,43] with adjustments as detailed previously [44,45]. The normalisation and statistical analyses were performed in Partek Genomics Suite Software version 6.5 (Partek Incorporated, St Louis, MO, USA). Array quality controls were performed by spike-in analysis of standard amounts of bacterial specific cDNA against respective homologous probe sets on the chip. Statistical differences between treatment groups were determined by one-way ANOVA with FDR tests for multiple comparisons. The fold change in gene expression was determined from the non log-transformed signal data after correction and normalisation. The experimental details and array CEL data files have been deposited under series name GSE42535 in NCBI’s Gene Expression Omnibus (GEO) database.

### Network and functional analysis

The groups of differentially expressed genes were uploaded into the Ingenuity Pathway Analysis (IPA) database for network and pathway determination (Ingenuity Systems, 2005). These datasets were also characterised according to their association with Gene Ontology (GO) terms listed under biological process using Gene Ontology Enrichment Analysis Software Toolkit (GOEAST) [46].

## Abbreviations

FSH: Follicle-stimulating hormone; LH: Luteinising hormone; TGFβ: Transforming growth factor beta; TNFα: Tumour necrosis factor alpha; qRT-PCR: Quantitative reverse transcription polymerase chain reaction; PCA: Principal component analysis; FDR: Benjamini-Hochberg false discovery rate; GO: Gene ontology; GOEAST: Gene ontology enrichment analysis software toolkit; GEO: Gene expression omnibus; IPA: Ingenuity pathway analysis; NCBI: National centre for biotechnology information; RMA: Robust multi-array average; LSD: Least significant difference; CV: Coefficient of variation.

## Competing interests

The authors declare that they have no competing interests.

## Authors’ contributions

Conceived and designed the experiments: PGK, CG and RJR. Performed the experiments: CG, PGK and RJR. Analysed the data: NH, KH, CG, PGK and RJR. Wrote the paper: NH, KH, CG, PGK and RJR. All authors read and approved the final manuscript.

## Supplementary Material

Additional file 1: Figure S1 Unsupervised hierarchical clustering across all probe sets (n = 24,182) for 16 arrays using the Euclidian dissimilarity algorithm with the average linkage method in Partek Genomics Suite. The heatmap represents the distribution of normalised signal intensity, grouped by pattern similarity for both probe set and array (indicated by dendrogram at top). Click here for file

Additional file 2: Figure S2 Principal component analyses of probe set intensity for all TNFα treatment arrays (n = 8) A, and FSH-treated and control arrays (n = 7) B. In A the arrays are numbered TNFα-treated (G3, G7, G11 and G15 in red) and TNFα plus FSH-treated (G4, G8, G12, and G16 in black). In B, the arrays are numbered (n = 4 per treatment) as follows: control granulosa (G1, G5, G9 and G13 in grey); FSH-treated (G2, G6, G10 and G14 in blue). The graph is a scatter plot of the values for the first (X) and second (Y) principal components based on the correlation matrix of the total normalised array intensity data. Click here for file

Additional file 3: Figure S3 Venn diagram of numbers of genes differentially regulated between TNFα ± FSH treatments and the control. Symbols indicate genes which are up (↑) and down regulated (↓). Click here for file

Additional file 4: Table S1 The numbers of probe sets which were differentially regulated between TNFα ± FSH treatments of cultured granulosa and the control. The numbers were determined by a one-way ANOVA with an FDR of *P* < 0.05 for multiple comparisons using Partek. Click here for file

Additional file 5: Table S2 A list of probe sets (n = 527) which are 3-fold differentially regulated between control (± FSH) (n = 7) and TNFα (± FSH)-treated (n = 8) groups with an FDR of *P* < 0.05 for multiple comparisons. The probe sets are listed in alphabetical order based on gene symbol, and those which have not been assigned a gene annotation have been placed at the end of the list. Click here for file
