# Supplementary figures and images for: The global effect of follicle-stimulating hormone and tumour necrosis factor α on gene expression in cultured bovine ovarian granulosa cells
Source: BMC Genomics. 2014 Jan 28;15:72. doi: 10.1186/1471-2164-15-72 (PMC3906957; doi:10.1186/1471-2164-15-72)

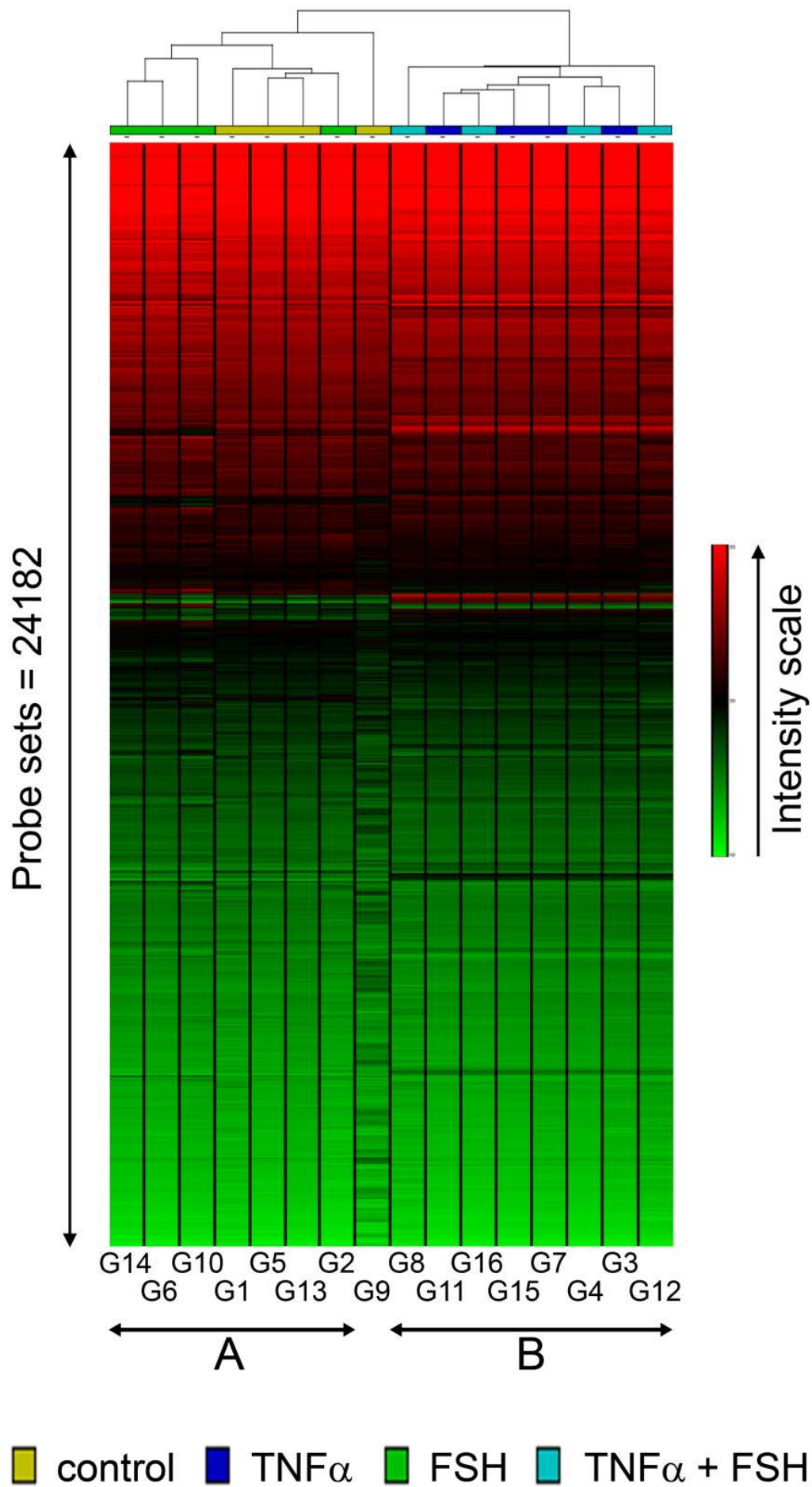

Supplement: Additional file 1: Figure S1 — Unsupervised hierarchical clustering across all probe sets (n = 24,182) for 16 arrays using the Euclidian dissimilarity algorithm with the average linkage method in Partek Genomics Suite. The heatmap represents the distribution of normalised signal intensity, grouped by pattern similarity for both probe set and array (indicated by dendrogram at top). [file 1471-2164-15-72-S1.pdf]

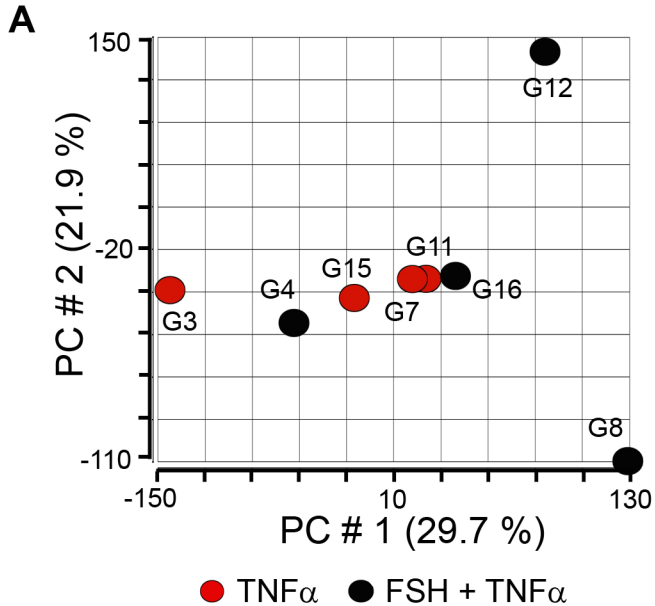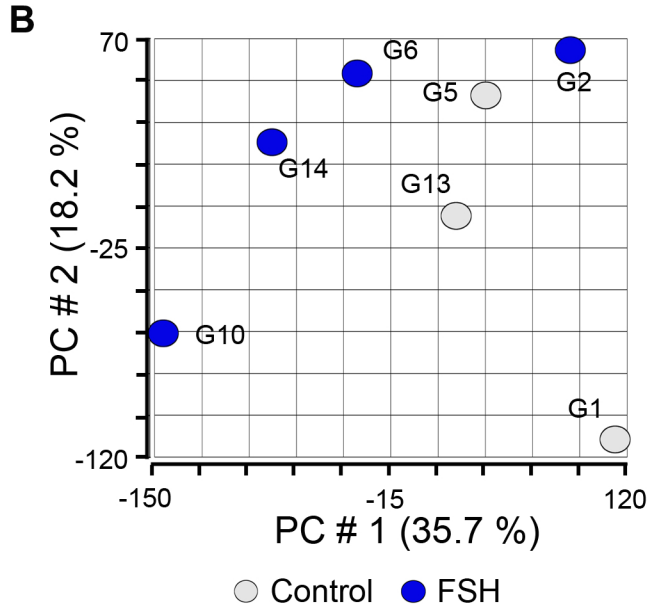

Supplement: Additional file 2: Figure S2 — Principal component analyses of probe set intensity for all TNFα treatment arrays (n = 8) A, and FSH-treated and control arrays (n = 7) B. In A the arrays are numbered TNFα-treated (G3, G7, G11 and G15 in red) and TNFα plus FSH-treated (G4, G8, G12, and G16 in black). In B, the arrays are numbered (n = 4 per treatment) as follows: control granulosa (G1, G5, G9 and G13 in grey); FSH-treated (G2, G6, G10 and G14 in blue). The graph is a scatter plot of the values for the first (X) and second (Y) principal components based on the correlation matrix of the total normalised array intensity data. [file 1471-2164-15-72-S2.pdf]
