# Supplementary material for: The global effect of follicle-stimulating hormone and tumour necrosis factor α on gene expression in cultured bovine ovarian granulosa cells
Source: BMC Genomics. 2014 Jan 28;15:72. doi: 10.1186/1471-2164-15-72 (PMC3906957; doi:10.1186/1471-2164-15-72)

Genes differentially regulated between TNFα treatments and control

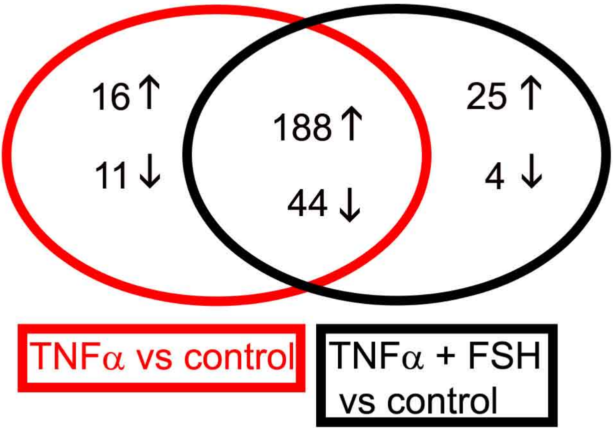

Supplement: Additional file 3: Figure S3 — Venn diagram of numbers of genes differentially regulated between TNFα ± FSH treatments and the control. Symbols indicate genes which are up (↑) and down regulated (↓). [file 1471-2164-15-72-S3.pdf]
