# Supplementary material for: The global effect of follicle-stimulating hormone and tumour necrosis factor α on gene expression in cultured bovine ovarian granulosa cells
Source: BMC Genomics. 2014 Jan 28;15:72. doi: 10.1186/1471-2164-15-72 (PMC3906957; doi:10.1186/1471-2164-15-72)
Supplement: Additional file 4: Table S1 — The numbers of probe sets which were differentially regulated between TNFα ± FSH treatments of cultured granulosa and the control. The numbers were determined by a one-way ANOVA with an FDR of P < 0.05 for multiple comparisons using Partek. [file 1471-2164-15-72-S4.pdf]

Treatments	Fold Change	Up Regulated	Down Regulated	Total
TNFα-treated (n = 4) versus control (n = 3)	>2	736	370	1106
	>3	360	97	457
	>4	211	35	246
	>6	105	5	110
	>10	36	0	36
TNFα + FSH-treated (n = 4) versus control (n = 3)	>2	741	395	1136
	>3	359	93	452
	>4	207	29	236
	>6	94	4	98
	>10	30	0	30
