# Supplementary material for: The global effect of follicle-stimulating hormone and tumour necrosis factor α on gene expression in cultured bovine ovarian granulosa cells
Source: BMC Genomics. 2014 Jan 28;15:72. doi: 10.1186/1471-2164-15-72 (PMC3906957; doi:10.1186/1471-2164-15-72)
Supplement: Additional file 5: Table S2 — A list of probe sets (n = 527) which are 3-fold differentially regulated between control (± FSH) (n = 7) and TNFα (± FSH)-treated (n = 8) groups with an FDR of P < 0.05 for multiple comparisons. The probe sets are listed in alphabetical order based on gene symbol, and those which have not been assigned a gene annotation have been placed at the end of the list. [file 1471-2164-15-72-S5.pdf]

Probeset ID	Gene Symbol	Gene Title	Fold Change (TNFα ± FSH versus control ± FSH)
Bt.23514.1.S1_at	A2M	alpha-2-macroglobulin	5.6
Bt.9593.1.S1_at	ABCC3	ATP-binding cassette, sub-family C (CFTR/MRP), member 3	8.0
Bt.24941.1.S1_at	ABHD3	abhydrolase domain containing 3	5.2
Bt.26223.1.S1_at	ABTB2	Ankyrin repeat and BTB (POZ) domain containing 2	3.2
Bt.9714.1.S1_at	ACTG2	actin, gamma 2, smooth muscle, enteric	-3.3
Bt.25469.1.A1_at	ADAM23	ADAM metallopeptidase domain 23	8.3
Bt.20236.1.S1_at	ADAMTSL4	ADAMTS-like 4	3.8
Bt.18840.1.S1_at	ADCY2	adenylate cyclase 2 (brain)	4.7
Bt.9697.1.A1_at	ADH6	alcohol dehydrogenase 6 (class V)	4.5
Bt.15735.1.S1_at	AGRN	agrin	3.8
Bt.8798.2.S1_at	ALDH1A3 /// LOC534200	aldehyde dehydrogenase 1 family, member A3 /// aldehyde dehydrogenase family 1, subfamily A3	5.7
Bt.8798.1.S1_at	ALDH1A3 /// LOC534200	aldehyde dehydrogenase 1 family, member A3 /// aldehyde dehydrogenase family 1, subfamily A3	11.7
Bt.7050.1.S1_at	AMH	anti-Mullerian hormone	-3.4
Bt.137.1.S1_at	ANGPT2	angiopoietin 2	-3.8
Bt.26461.1.A1_at	ANKH	ankylosis, progressive homolog (mouse)	3.7
Bt.5026.1.S1_at	ANXA8L1	annexin A8-like 1	8.0
Bt.9267.1.A1_at	APOBEC3B	apolipoprotein B mRNA editing enzyme, catalytic polypeptide-like 3B	3.9
Bt.3313.1.A1_at	ARHGAP24	Rho GTPase activating protein 24	4.8
Bt.12603.1.S1_at	ARHGAP29	Rho GTPase activating protein 29	7.1
Bt.21143.1.A1_at	ARHGAP29	Rho GTPase activating protein 29	8.7
Bt.4757.1.S1_at	ARHGDIB	Rho GDP dissociation inhibitor (GDI) beta	3.8

Bt.24442.1.A1_at	ARHGEF11	Rho guanine nucleotide exchange factor (GEF) 11	5.1
Bt.18132.1.A1_at	ARHGEF5 /// LOC787930	Rho guanine nucleotide exchange factor (GEF) 5 /// Temporarily Assigned Gene name family	13.6
Bt.26760.1.S1_at	ARRDC2	arrestin domain containing 2	4.3
Bt.29440.1.S1_at	ATP4B	ATPase, H+/K+ exchanging, beta polypeptide	-3.1
Bt.9004.1.S1_at	BCAT2	branched chain amino-acid transaminase 2, mitochondrial	4.3
Bt.7542.1.S1_at	BCL2A1	BCL2-related protein A1	5.3
Bt.16678.1.S1_at	BEX2	brain expressed X-linked 2	8.6
Bt.23123.1.S1_at	BHLHE40	basic helix-loop-helix family, member e40	4.8
Bt.22344.1.S1_at	BHLHE40	basic helix-loop-helix family, member e40	4.1
Bt.9391.1.A1_at	BIRC3	baculoviral IAP repeat-containing 3	5.1
Bt.9391.2.S1_at	BIRC3	baculoviral IAP repeat-containing 3	8.6
Bt.2592.1.A1_x_at	BOLA	MHC class I heavy chain	5.0
Bt.8121.1.S1_x_at	BOLA	MHC class I heavy chain	3.2
Bt.27283.1.S1_x_at	BOLA	MHC class I heavy chain	3.6
Bt.29815.1.S1_x_at	BOLA	MHC class I heavy chain	4.9
Bt.29824.1.S1_s_at	BOLA /// BOLA	MHC class I heavy chain /// MHC class I antigen clone 2	6.6
Bt.4762.1.S1_at	BOLA /// BOLA /// BOLA-NC1 /// JSP.1	MHC class I heavy chain /// MHC class I antigen clone 2 /// non-classical MHC class I a	3.2
Bt.27760.1.S1_at	BoLA /// BOLA-A	major histocompatibility complex, class I, A /// major histocompatibility complex	5.7
Bt.29814.1.S1_at	BoLA /// BOLA-A /// LOC505676	major histocompatibility complex, class I, A /// major histocompatibility complex	5.9
Bt.1007.1.S1_at	BOLA-DMB	major histocompatibility complex, class II, DM beta-chain, expressed	5.0
Bt.22867.1.S1_x_at	BOLA-DQA1	major histocompatibility complex, class II, DQ alpha, type 1	3.0
Bt.22867.2.A1_at	BOLA-DQA1	major histocompatibility complex, class II, DQ alpha, type 1	3.7

Bt.4751.1.S1_a_at	BOLA-DQA2	major histocompatibility complex, class II, DQ alpha 2	5.9
Bt.8552.1.S1_at	BOLA-DRA	major histocompatibility complex, class II, DR alpha	4.6
Bt.5356.1.S1_s_at	BoLA-DRB3	major histocompatibility complex, class II, DRB3	3.3
Bt.3805.1.S1_at	BOLA-N /// JSP.1	MHC class I antigen /// MHC Class I JSP.1	4.4
Bt.28022.1.A1_s_at	BOLA-NC1	Non-classical MHC class I antigen	3.3
Bt.19795.1.S1_at	BREH1	retinyl ester hydrolase type 1	3.3
Bt.15788.3.S1_at	BST2	Bone marrow stromal cell antigen 2	6.1
Bt.15788.1.S1_a_at	BST2	Bone marrow stromal cell antigen 2	6.4
Bt.1491.1.S1_at	C1S	complement component 1, s subcomponent	3.6
Bt.6731.1.S1_at	C3H1orf54	chromosome 1 open reading frame 54 ortholog	3.9
Bt.22854.1.S1_at	CA2	carbonic anhydrase II	4.4
Bt.26040.1.A1_at	CA5B	carbonic anhydrase VB, mitochondrial	3.8
Bt.22726.1.A1_at	CA8	Carbonic anhydrase VIII	3.1
Bt.5718.1.S1_at	CACNA1G	calcium channel, voltage-dependent, T type, alpha 1G subunit	6.9
Bt.17491.1.A1_at	CAMK1D	calcium/calmodulin-dependent protein kinase ID	3.3
Bt.16175.1.A1_at	CAPRIN2	caprin family member 2	-3.5
Bt.16018.1.S1_a_at	CASP4	caspase 4, apoptosis-related cysteine peptidase	15.2
Bt.16018.1.S2_at	CASP4	caspase 4, apoptosis-related cysteine peptidase	10.8
Bt.2230.1.S1_at	CAV1	caveolin 1, caveolae protein, 22kDa	-4.0
Bt.2230.1.S2_at	CAV1	caveolin 1, caveolae protein, 22kDa	-4.1
Bt.3478.1.A1_s_at	CAV1	caveolin 1, caveolae protein, 22kDa	-3.5
Bt.21095.1.S1_at	CCDC85C	coiled-coil domain containing 85C	3.4
Bt.2408.1.S1_s_at	CCL2	chemokine (C-C motif) ligand 2	11.6
Bt.2408.1.S1_at	CCL2	chemokine (C-C motif) ligand 2	13.1
Bt.552.1.S1_at	CCL5	chemokine (C-C motif) ligand 5	6.4

Bt.4895.1.S1_at	CCND2	cyclin D2	-3.1
Bt.13384.1.S1_at	CD19 /// LOC100298507	CD19 molecule /// hypothetical protein LOC100298507	14.2
Bt.5861.1.S1_at	CD200	CD200 molecule	10.8
Bt.5392.1.S1_at	CD36	CD36 molecule (thrombospondin receptor)	-3.6
Bt.13130.1.S1_at	CD40	CD40 molecule, TNF receptor superfamily member 5	10.1
Bt.5494.1.S1_at	CD44	CD44 molecule (Indian blood group)	21.5
Bt.21980.1.S1_at	CD72	CD72 molecule	3.1
Bt.12443.1.S1_at	CD82 /// LOC100335281	CD82 molecule /// CD82 molecule-like	4.8
Bt.3841.2.S1_at	CD83	CD83 molecule	3.8
Bt.3841.1.S1_at	CD83	CD83 molecule	6.3
Bt.19561.1.S1_at	CD83	CD83 molecule	6.0
Bt.2573.1.S1_at	CD9	CD9 molecule	3.6
Bt.5168.1.S1_at	CDC42EP1	CDC42 effector protein (Rho GTPase binding) 1	3.3
Bt.11241.1.S1_at	CDH1	cadherin 1, type 1, E-cadherin (epithelial)	3.1
Bt.10027.2.S1_a_at	CEACAM8	carcinoembryonic antigen-related cell adhesion molecule 8	3.2
Bt.10027.1.S2_at	CEACAM8	carcinoembryonic antigen-related cell adhesion molecule 8	3.1
Bt.4336.1.S1_at	CFD	complement factor D (adipsin)	3.1
Bt.5084.1.S1_at	CKB	creatine kinase, brain	3.4
Bt.3885.5.S1_x_at	CLCA3P	chloride channel accessory 3 (pseudogene)	3.0
Bt.16297.1.S1_at	CLDND1	claudin domain containing 1	-3.3
Bt.3881.1.S1_at	CNGA3	cyclic nucleotide gated channel alpha 3	4.3
Bt.20512.2.S1_at	COL15A1	collagen, type XV, alpha 1	4.5
Bt.20512.1.S1_at	COL15A1	collagen, type XV, alpha 1	9.4
Bt.12697.1.S1_a_at	COL16A1	collagen, type XVI, alpha 1	5.0
Bt.12697.1.S1_at	COL16A1	collagen, type XVI, alpha 1	3.4
Bt.11570.1.A1_at	COL5A3	Collagen, type V, alpha 3	4.6

Bt.23508.1.A1_at	COL6A1	collagen, type VI, alpha 1	14.5
Bt.13681.2.S1_at	COL6A1	collagen, type VI, alpha 1	3.1
Bt.4056.1.S1_at	CRIP2	cysteine-rich protein 2	3.2
Bt.78.1.S1_at	CRYBB1	crystallin, beta B1	4.4
Bt.2262.1.S1_at	CSRP2	cysteine and glycine-rich protein 2	3.1
Bt.24354.1.S1_at	CSTB	cystatin B (stefin B)	3.1
Bt.3251.1.S1_at	CTNNAL1	catenin (cadherin-associated protein), alpha-like 1	-3.3
Bt.20694.1.A1_at	CTNNAL1	catenin (cadherin-associated protein), alpha-like 1	-3.2
Bt.1031.1.S1_at	CTSH	cathepsin H	3.5
Bt.16966.1.S1_at	CXCL10	chemokine (C-X-C motif) ligand 10	10.8
Bt.22000.1.A1_at	CYR61	cysteine-rich, angiogenic inducer, 61	-4.9
Bt.3814.1.S1_at	DAB2	disabled homolog 2, mitogen-responsive phosphoprotein (Drosophila)	-3.4
Bt.2726.2.S1_at	DACT1	dapper, antagonist of beta-catenin, homolog 1 (Xenopus laevis)	-3.6
Bt.2726.1.S1_at	DACT1	dapper, antagonist of beta-catenin, homolog 1 (Xenopus laevis)	-4.1
Bt.24335.1.S1_at	DAPP1	dual adaptor of phosphotyrosine and 3-phosphoinositides	5.0
Bt.16272.1.S1_at	DCLK1	doublecortin-like kinase 1	4.5
Bt.6933.1.S1_at	DCLK1	Doublecortin-like kinase 1	3.8
Bt.16272.2.A1_at	DCLK1	doublecortin-like kinase 1	4.2
Bt.23651.1.A1_at	DDAH1	dimethylarginine dimethylaminohydrolase 1	-4.2
Bt.115.1.S1_at	DDC	dopa decarboxylase (aromatic L-amino acid decarboxylase)	8.7
Bt.24033.1.A1_at	DDX58	DEAD (Asp-Glu-Ala-Asp) box polypeptide 58	3.3
Bt.19813.1.A1_at	DNER	delta/notch-like EGF repeat containing	5.8
Bt.24191.1.A1_at	DPY19L1	Dpy-19-like 1 (C. elegans)	3.6
Bt.18685.1.A1_at	DTX1	deltex homolog 1 (Drosophila)	3.3
Bt.26867.2.S1_a_at	EEPD1	endonuclease/exonuclease/phosphatase family domain containing 1	-4.4

Bt.5056.2.A1_at	EFEMP1	EGF-containing fibulin-like extracellular matrix protein 1	-3.2
Bt.19309.1.S1_at	EFNA5	ephrin-A5	-6.5
Bt.3278.1.A1_at	EFNA5	ephrin-A5	-7.3
Bt.19309.3.A1_at	EFNA5	ephrin-A5	-6.4
Bt.23232.1.S1_at	EGLN3	egl nine homolog 3 (C. elegans)	4.5
Bt.12875.1.S1_at	EMP3	epithelial membrane protein 3	3.3
Bt.21220.1.S1_at	EMR3	egf-like module-containing mucin-like receptor 3	3.5
Bt.16356.1.S1_at	ENTPD3	Ectonucleoside triphosphate diphosphohydrolase 3	17.7
Bt.24404.1.A1_at	EPB41L3	erythrocyte membrane protein band 4.1-like 3	6.3
Bt.25447.1.A1_at	EPHA7	EPH receptor A7	-3.6
Bt.9500.1.S1_a_at	EPSTI1	epithelial stromal interaction 1 (breast)	3.9
Bt.25649.1.A1_at	ERAP2	endoplasmic reticulum aminopeptidase 2	3.8
Bt.25738.1.A1_at	ERP27	endoplasmic reticulum protein 27	3.5
Bt.5914.1.S1_at	FATE1	fetal and adult testis expressed 1	6.2
Bt.6449.1.S1_at	FBLN5	fibulin 5	-4.8
Bt.13428.2.S1_at	FBP1	fructose-1,6-bisphosphatase 1	3.3
Bt.25107.1.A1_at	FGD5	FYVE, RhoGEF and PH domain containing 5	-3.2
Bt.15864.1.S1_at	FGF2	fibroblast growth factor 2 (basic)	3.2
Bt.1411.1.S1_at	FMNL3	formin-like 3	3.4
Bt.3797.1.S1_at	FMOD	fibromodulin	4.0
Bt.23418.1.S1_at	FN1	fibronectin 1	3.3
Bt.27526.1.A1_at	FOXA3	forkhead box A3	9.0
Bt.24663.1.A1_at	FOXS1	forkhead box S1	3.3
Bt.405.1.S1_at	FST	follistatin	-6.2
Bt.9573.1.S1_a_at	FXYD3	FXYD domain containing ion transport regulator 3	3.9
Bt.176.1.S1_at	GAL	galanin prepropeptide	4.6

Bt.28744.1.S1_at	GBP4	guanylate binding protein 4	4.8
Bt.16350.2.A1_s_at	GBP5	guanylate binding protein 5	4.0
Bt.21773.2.S1_at	GBP5	guanylate binding protein 5	3.3
Bt.21773.1.A1_at	GBP5	guanylate binding protein 5	3.7
Bt.16068.1.A1_at	GCLC	Glutamate-cysteine ligase, catalytic subunit	-3.4
Bt.28572.1.S1_at	GFI1	growth factor independent 1 transcription repressor	14.1
Bt.29262.1.S1_at	GKN2	gastrokine 2	3.4
Bt.13486.1.A1_at	GLDC	glycine dehydrogenase (decarboxylating)	3.8
Bt.13486.2.S1_at	GLDC	Glycine dehydrogenase (decarboxylating)	5.2
Bt.14464.1.A1_at	GPHN	gephyrin	3.0
Bt.22504.2.S1_at	GPR56	G protein-coupled receptor 56	3.1
Bt.4597.1.S1_at	GPR68	G protein-coupled receptor 68	5.2
Bt.26157.1.A1_at	GPR77	G protein-coupled receptor 77	61.5
Bt.3178.1.A1_at	GRIA3	glutamate receptor, ionotrophic, AMPA 3	3.7
Bt.20280.1.S1_at	GYLTL1B	glycosyltransferase-like 1B	-3.6
Bt.5348.1.S1_at	H19	H19, imprinted maternally expressed transcript (non-protein coding)	3.5
Bt.24694.1.A1_at	HEG1	HEG homolog 1 (zebrafish)	-5.0
Bt.22498.2.S1_at	HES4	Hairy and enhancer of split 4 (Drosophila)	4.6
Bt.20348.1.A1_at	HEYL	hairy/enhancer-of-split related with YRPW motif-like	4.7
Bt.9226.1.S1_at	HPCAL1	hippocalcin-like 1	4.2
Bt.8127.1.S2_at	HPSE	heparanase	-4.1
Bt.8127.1.S1_at	HPSE	heparanase	-3.7
Bt.13027.1.A1_at	HSD11B1	hydroxysteroid (11-beta) dehydrogenase 1	7.0
Bt.22879.1.S1_at	HSD17B1	hydroxysteroid (17-beta) dehydrogenase 1	-4.8
Bt.7478.1.S1_at	HSPB6	heat shock protein, alpha-crystallin-related, B6	3.8

Bt.5372.1.S1_at	ICAM1	intercellular adhesion molecule 1	4.7
Bt.27759.2.S1_at	IDO1	indoleamine 2,3-dioxygenase 1	7.4
Bt.27759.1.A1_at	IDO1	indoleamine 2,3-dioxygenase 1	7.7
Bt.2153.1.S1_at	IER3	immediate early response 3	3.6
Bt.22021.1.S1_at	IFI16	interferon, gamma-inducible protein 16	13.6
Bt.11259.1.S1_at	IFI27	putative ISG12(a) protein	9.5
Bt.1548.1.S1_at	IFI30	gamma-inducible protein 30	3.2
Bt.19620.1.A1_at	IFI44	interferon-induced protein 44	7.4
Bt.20785.2.S1_at	IFI44 /// LOC781857	interferon-induced protein 44 /// histocompatibility 28-like	14.9
Bt.20785.1.A1_at	IFI44 /// LOC781857	interferon-induced protein 44 /// histocompatibility 28-like	10.6
Bt.17729.1.A1_at	IFI44 /// LOC781857	interferon-induced protein 44 /// histocompatibility 28-like	11.0
Bt.8436.1.S1_at	IFI6	interferon, alpha-inducible protein 6	9.2
Bt.16857.1.A1_at	IFIH1	interferon induced with helicase C domain 1	7.1
Bt.24098.1.A1_at	IFIH1	interferon induced with helicase C domain 1	6.2
Bt.24795.1.A1_at	IFIT2	interferon-induced protein with tetratricopeptide repeats 2	6.0
Bt.22978.1.S1_a_at	IFITM1	interferon induced transmembrane protein 1 (9-27)	3.3
Bt.22978.2.S1_at	IFITM1	interferon induced transmembrane protein 1 (9-27)	3.4
Bt.23176.2.S1_a_at	IGF2	insulin-like growth factor 2 (somatomedin A)	3.1
Bt.20359.1.S1_at	IKZF3	IKAROS family zinc finger 3 (Aiolos)	3.8
Bt.15910.1.S1_at	IL1F6	interleukin 1 family, member 6 (epsilon)	3.6
Bt.9175.1.A1_at	IL1R1	interleukin 1 receptor, type I	4.5
Bt.4199.1.S1_at	IL1RN	interleukin 1 receptor antagonist	5.9
Bt.4897.1.S1_at	INHA	inhibin, alpha	-5.8
Bt.12760.1.S1_at	INHBA	inhibin, beta A	-5.8
Bt.10077.1.S2_at	IRF1	interferon regulatory factor 1	4.7
Bt.10077.1.S3_at	IRF1	interferon regulatory factor 1	3.2

Bt.13902.1.S1_at	IRF8	interferon regulatory factor 8	4.2
Bt.12304.1.S1_at	ISG15	ISG15 ubiquitin-like modifier	10.8
Bt.22275.1.A1_at	ISG20	interferon stimulated exonuclease gene 20kDa	4.0
Bt.15707.1.S1_at	ITGA5	integrin, alpha 5 (fibronectin receptor, alpha polypeptide)	4.4
Bt.6125.1.S1_at	JAG1	jagged 1	6.7
Bt.6125.2.S1_at	JAG1	jagged 1	6.1
Bt.546.2.S1_at	KCNMA1	potassium large conductance calcium-activated channel, subfamily M, alpha member 1	3.4
Bt.546.1.S1_at	KCNMA1	potassium large conductance calcium-activated channel, subfamily M, alpha member 1	8.9
Bt.28008.1.S1_s_at	KCNN2	potassium intermediate/small conductance calcium-activated channel, subfamily N, member	-3.6
Bt.26445.1.A1_at	KIT	v-kit Hardy-Zuckerman 4 feline sarcoma viral oncogene homolog	-3.0
Bt.1745.1.S1_at	KRT18	keratin 18	4.2
Bt.23608.1.S1_s_at	KRT8	keratin 8	10.0
Bt.1416.1.S1_at	LGALS3	lectin, galactoside-binding, soluble, 3	-3.1
Bt.27564.1.A1_at	LGP2	RNA helicase LGP2	4.4
Bt.17570.2.A1_at	LINGO2	leucine rich repeat and Ig domain containing 2	-3.7
Bt.26232.1.S1_at	LOC100296192	similar to nuclear antigen Sp100	4.5
Bt.24813.3.S1_at	LOC100335957 /// RTP4	28kD interferon responsive protein-like /// receptor (chemosensory) transporter protein	7.1
Bt.24813.2.S1_at	LOC100335957 /// RTP4	28kD interferon responsive protein-like /// receptor (chemosensory) transporter protein	6.2
Bt.24813.1.A1_at	LOC100335957 /// RTP4	28kD interferon responsive protein-like /// receptor (chemosensory) transporter protein	14.5
Bt.28177.1.A1_at	LOC100335974	multiple C2 domains, transmembrane 2	6.6
Bt.1938.1.S1_at	LOC100336454	hypothetical protein LOC100336454	8.4

Bt.28344.1.S1_at	LOC507695	hypothetical LOC507695	3.4
Bt.25111.1.A1_at	LOC508347	interferon-induced protein 44-like	10.9
Bt.24112.1.A1_at	LOC511907	similar to Chromosome X open reading frame 57	5.6
Bt.10124.1.S1_at	LOC513129	hypothetical LOC513129	3.5
Bt.21820.1.S1_at	LOC530077	similar to GTPase, IMAP family member 5	6.5
Bt.26568.2.S1_a_at	LOC531049	similar to Putative eukaryotic translation initiation factor 3 subunit (eIF-3)	4.0
Bt.26568.1.S1_a_at	LOC531049	similar to Putative eukaryotic translation initiation factor 3 subunit (eIF-3)	3.9
Bt.1785.1.A1_at	LOC532189	carboxypeptidase D-like	-3.3
Bt.19937.2.S1_at	LOC532189	carboxypeptidase D-like	-3.2
Bt.12405.1.S1_at	LOC538276	similar to Janus kinase 3	-4.4
Bt.15840.1.A1_at	LOC539889	inositol polyphosphate-5-phosphatase F	6.2
Bt.11916.1.S1_at	LOC615412	BAI1-associated protein 2-like 1-like	3.7
Bt.19174.1.A1_at	LOC786550	GCL (Drosophila germ cell-less) homolog family member (gcl-1)-like	3.6
Bt.23205.1.S1_s_at	LOC787239	DKFZP459P193 protein-like	-4.2
Bt.4641.1.S1_at	LOC789476 /// STAT5A	signal transducer and activator of transcription 5B /// signal transducer and activator	3.1
Bt.12297.1.S1_at	LOXL4	lysyl oxidase-like 4	8.8
Bt.8135.1.S1_at	LRAT	lecithin retinol acyltransferase (phosphatidylcholine--retinol O-acyltransferase)	3.5
Bt.4108.1.S1_at	LRRFIP1	leucine rich repeat (in FLII) interacting protein 1	4.0
Bt.23267.1.S1_at	LYVE1	lymphatic vessel endothelial hyaluronan receptor 1	3.6
Bt.24309.1.A1_at	LYVE1	lymphatic vessel endothelial hyaluronan receptor 1	4.6
Bt.28686.1.S1_at	MAL2	mal, T-cell differentiation protein 2	-4.0
Bt.13769.1.S1_at	MAP2	Microtubule-associated protein 2	3.2

Bt.21934.1.S1_a_at	MAP2	Microtubule-associated protein 2	4.7
Bt.3092.1.S1_at	MFAP2	microfibrillar-associated protein 2	3.0
Bt.13235.1.S1_at	MGC148992	similar to RGC-32	3.2
Bt.27866.1.A1_at	MIOX	myo-inositol oxygenase	4.7
Bt.9774.1.S1_a_at	MIR147	microRNA mir-147	5.3
Bt.5313.1.S1_at	MMP2	matrix metallopeptidase 2 (gelatinase A, 72kDa gelatinase, 72kDa type IV collagenase)	5.0
Bt.9605.1.S1_at	MS4A8B	membrane-spanning 4-domains, subfamily A, member 8B	4.6
Bt.9207.1.S1_at	MSN	moesin	3.4
Bt.26959.2.S1_at	MVK	mevalonate kinase	-3.7
Bt.4675.1.S1_a_at	MX1	myxovirus (influenza virus) resistance 1, interferon-inducible protein p78 (mouse)	6.6
Bt.8143.1.S1_at	MX2	myxovirus (influenza virus) resistance 2 (mouse)	7.5
Bt.12781.2.S1_a_at	MYB	v-myb myeloblastosis viral oncogene homolog (avian)	6.1
Bt.12781.1.S1_at	MYB	v-myb myeloblastosis viral oncogene homolog (avian)	8.4
Bt.979.1.A1_at	MYO1B	myosin IB	-3.1
Bt.10999.2.S1_at	NCK2	NCK adaptor protein 2	3.1
Bt.8804.1.S1_at	NELL2	NEL-like 2 (chicken)	3.5
Bt.20288.1.S1_at	NFKB2	nuclear factor of kappa light polypeptide gene enhancer in B-cells 2 (p49/p100)	3.7
Bt.9027.1.S1_at	NFKBIA	nuclear factor of kappa light polypeptide gene enhancer in B-cells inhibitor, alpha	9.8
Bt.8227.1.S1_at	NFKBIZ	nuclear factor of kappa light polypeptide gene enhancer in B-cells inhibitor, zeta	3.6
Bt.26636.1.S1_at	NKG7	natural killer cell group 7 sequence	3.4
Bt.10630.1.S1_at	NMB	neuromedin B	-5.5
Bt.17865.1.A1_at	NOSTRIN	nitric oxide synthase trafficker	3.4

Bt.7157.1.S1_at	NPC1	Niemann-Pick disease, type C1	-3.0
Bt.7393.1.S1_at	NPNT	Nephronectin	-4.6
Bt.10060.1.S1_at	NR4A2	nuclear receptor subfamily 4, group A, member 2	4.3
Bt.10845.1.S1_at	NR5A2	nuclear receptor subfamily 5, group A, member 2	-3.1
Bt.411.1.S1_at	NRG1	neuregulin 1	3.9
Bt.22873.1.S1_at	NTRK1	neurotrophic tyrosine kinase, receptor, type 1	3.6
Bt.440.1.S1_at	NTS	neurotensin	-3.2
Bt.20360.1.A1_at	NUAK2	NUAK family, SNF1-like kinase, 2	3.4
Bt.4167.1.S1_at	NUCB2	nucleobindin 2	5.2
Bt.23597.1.S1_at	NUPR1	nuclear protein, transcriptional regulator, 1	4.0
Bt.11030.1.S1_at	OAF	OAF homolog (Drosophila)	3.2
Bt.20922.1.S1_at	OAS1	2',5'-oligoadenylate synthetase 1, 40/46kDa	5.5
Bt.20891.1.S1_at	OAS2	2'-5'-oligoadenylate synthetase 2, 69/71kDa	10.5
Bt.367.1.S1_at	OLR1	oxidized low density lipoprotein (lectin-like) receptor 1	4.0
Bt.24157.1.A1_at	PARM1	prostate androgen-regulated mucin-like protein 1	6.5
Bt.18116.3.A1_s_at	PARP12	poly (ADP-ribose) polymerase family, member 12	3.7
Bt.16376.1.S1_at	PARP12	poly (ADP-ribose) polymerase family, member 12	3.3
Bt.14676.1.S1_at	PARP14	poly (ADP-ribose) polymerase family, member 14	3.7
Bt.20409.1.S1_at	PARP9	poly (ADP-ribose) polymerase family, member 9	3.2
Bt.20286.1.S1_at	PDE4B	phosphodiesterase 4B, cAMP-specific	3.5
Bt.27755.1.S1_at	PDGFB	platelet-derived growth factor beta polypeptide (simian sarcoma viral (v-sis) oncogene	3.8
Bt.13330.1.S1_at	PDK4	pyruvate dehydrogenase kinase, isozyme 4	-3.6
Bt.10813.1.S1_at	PDLIM4	PDZ and LIM domain 4	4.8
Bt.12295.1.S1_at	PDPN	podoplanin	3.5
Bt.13689.1.A1_at	PIK3CD	Phosphoinositide-3-kinase, catalytic, delta polypeptide	5.0

Bt.4063.1.S1_at	PLA2G7	phospholipase A2, group VII (platelet-activating factor acetylhydrolase, plasma)	-3.5
Bt.15912.1.S1_at	PLAC8	placenta-specific 8	7.7
Bt.1942.1.S1_at	PLAUR	plasminogen activator, urokinase receptor	3.8
Bt.6608.1.S1_at	PLXNC1	Plexin C1	-3.3
Bt.26968.1.S1_at	PLXNC1	plexin C1	-4.4
Bt.16053.1.S1_at	PLXND1	plexin D1	9.4
Bt.11304.1.S1_at	PNKD	paroxysmal nonkinesigenic dyskinesia	3.2
Bt.20893.1.A1_at	PNPO	pyridoxamine 5'-phosphate oxidase	5.0
Bt.20893.2.S1_at	PNPO	pyridoxamine 5'-phosphate oxidase	4.0
Bt.20372.1.S1_at	PON3	paraoxonase 3	9.3
Bt.23509.1.S1_at	PPAP2B	phosphatidic acid phosphatase type 2B	-5.5
Bt.3862.1.S1_a_at	PPARG	peroxisome proliferator-activated receptor gamma	-4.1
Bt.12250.1.S1_at	PPP2R3C	protein phosphatase 2, regulatory subunit B", gamma	4.3
Bt.5435.1.S1_at	PRKCB	protein kinase C, beta	15.7
Bt.14035.1.S1_at	PRKCB	protein kinase C, beta	19.3
Bt.22234.1.S1_at	PRKCQ	protein kinase C, theta	3.9
Bt.4404.1.A1_at	PRSS2	protease, serine, 2 (trypsin 2)	3.1
Bt.24364.1.A1_at	PRSS35	protease, serine, 35	-3.4
Bt.3624.1.S1_at	PSMB10	proteasome (prosome, macropain) subunit, beta type, 10	3.9
Bt.15865.1.S1_at	PSMB8	proteasome (prosome, macropain) subunit, beta type, 8 (large multifunctional peptidase	3.8
Bt.20532.1.S1_at	PSMB9	proteasome (prosome, macropain) subunit, beta type, 9 (large multifunctional peptidase	5.1
Bt.9662.1.S1_at	PTGER4	prostaglandin E receptor 4 (subtype EP4)	13.2
Bt.5247.1.S1_at	PTGIS	prostaglandin I2 (prostacyclin) synthase	12.2
Bt.15758.1.S1_at	PTGS2	prostaglandin-endoperoxide synthase 2 (prostaglandin G/H	-4.6

		synthase and cyclooxygenase)	
Bt.4844.1.S1_at	PTN	pleiotrophin	-3.5
Bt.11586.1.S1_at	PYCARD	PYD and CARD domain containing	3.3
Bt.14853.1.A1_at	RAB3IP	RAB3A interacting protein (rabin3)	3.8
Bt.26605.1.S1_at	RASAL1	RAS protein activator like 1 (GAP1 like)	5.2
Bt.1974.1.S1_at	RASL11B	RAS-like, family 11, member B	-3.2
Bt.20161.1.S1_at	RASSF4	Ras association (RalGDS/AF-6) domain family member 4	3.2
Bt.11525.2.A1_at	RBPMS	RNA binding protein with multiple splicing	3.9
Bt.11525.1.S1_a_at	RBPMS	RNA binding protein with multiple splicing	3.1
Bt.28209.1.S1_at	RGMB	RGM domain family, member B	-3.8
Bt.9487.1.S1_at	RGNEF	190 kDa guanine nucleotide exchange factor	-3.2
Bt.8752.1.S1_at	RGNEF	190 kDa guanine nucleotide exchange factor	-3.3
Bt.26612.2.S1_at	RGS16	regulator of G-protein signaling 16	4.1
Bt.10855.1.S1_at	RGS2	regulator of G-protein signaling 2, 24kDa	-4.6
Bt.13870.1.A1_at	RIPK4	receptor-interacting serine-threonine kinase 4	3.2
Bt.27401.1.A1_at	RNF128	ring finger protein 128	3.0
Bt.23672.1.A1_at	ROBO1	roundabout, axon guidance receptor, homolog 1 (Drosophila)	6.1
Bt.4177.3.A1_at	ROBO2	roundabout, axon guidance receptor, homolog 2 (Drosophila)	-7.9
Bt.24467.1.S1_at	RSAD2	radical S-adenosyl methionine domain containing 2	4.3
Bt.278.1.S1_at	SAA3	serum amyloid A 3	4.6
Bt.28624.1.S1_at	SAMD9	Sterile alpha motif domain containing 9	4.9
Bt.21116.1.S1_at	SELENBP1	selenium binding protein 1	4.4
Bt.2712.1.S1_at	SERPINA5	serpin peptidase inhibitor, clade A (alpha-1 antiproteinase, antitrypsin), member 5	12.5
Bt.13073.1.A1_at	SERPINB2	serpin peptidase inhibitor, clade B (ovalbumin), member 2	5.4
Bt.13073.1.S1_at	SERPINB2	serpin peptidase inhibitor, clade B (ovalbumin), member 2	14.9

Bt.12506.1.S1_at	SERPINE2	serpin peptidase inhibitor, clade E (nexin, plasminogen activator inhibitor type 1), me	-3.0
Bt.11057.1.S1_at	SHISA3	shisa homolog 3 (Xenopus laevis)	4.1
Bt.12636.1.A1_at	SLAMF8	SLAM family member 8	7.4
Bt.2989.2.S1_at	SLC25A12	solute carrier family 25 (mitochondrial carrier, Aralar), member 12	3.0
Bt.3964.1.S1_at	SLC2A3	solute carrier family 2 (facilitated glucose transporter), member 3	-3.7
Bt.4614.1.S1_at	SLC37A1	solute carrier family 37 (glycerol-3-phosphate transporter), member 1	3.5
Bt.2990.1.S1_at	SLCO2A1	solute carrier organic anion transporter family, member 2A1	3.4
Bt.27240.2.S1_at	SLCO4A1	solute carrier organic anion transporter family, member 4A1	4.8
Bt.27240.1.A1_at	SLCO4A1	Solute carrier organic anion transporter family, member 4A1	12.3
Bt.15484.2.A1_at	SLPI	secretory leukocyte peptidase inhibitor	58.6
Bt.3604.1.A1_at	SMAD2	SMAD family member 2	-3.1
Bt.8491.1.S1_at	SMOC2	SPARC related modular calcium binding 2	-3.4
Bt.11271.1.S1_at	SMOC2	SPARC related modular calcium binding 2	-3.4
Bt.3046.1.S1_at	SNAI1	snail homolog 1 (Drosophila)	3.4
Bt.28589.1.S1_at	SORBS2	sorbin and SH3 domain containing 2	-4.5
Bt.5153.1.S1_at	SPARCL1	SPARC-like 1 (hevin)	5.4
Bt.2520.1.S1_at	SPOCK2	sparc/osteonectin, cwcv and kazal-like domains proteoglycan (testican) 2	-5.0
Bt.2632.1.S1_at	SPP1	secreted phosphoprotein 1	3.6
Bt.5296.1.S1_at	SQRDL	sulfide quinone reductase-like (yeast)	3.5
Bt.28761.1.A1_s_at	SQRDL	sulfide quinone reductase-like (yeast)	4.1
Bt.16048.1.S1_at	SRGN	serglycin	-5.5
Bt.10272.1.S1_at	STC1	stanniocalcin 1	-5.8

Bt.3704.3.A1_at	STMN2	stathmin-like 2	7.7
Bt.29837.1.S1_at	SUSD4	sushi domain containing 4	-4.5
Bt.21053.2.S1_at	SYNGR1	synaptogyrin 1	3.1
BtAffx.1.13.S1_at	TAC3	tachykinin 3	8.7
Bt.8220.1.A1_at	TACC1	transforming, acidic coiled-coil containing protein 1	3.1
Bt.20416.1.S1_at	TAP1	transporter 1, ATP-binding cassette, sub-family B (MDR/TAP)	3.5
Bt.6651.1.S1_at	TBX3	T-box 3	5.2
Bt.21037.1.S1_at	TCF7	transcription factor 7 (T-cell specific, HMG-box)	4.8
Bt.21037.2.S1_at	TCF7	transcription factor 7 (T-cell specific, HMG-box)	4.7
Bt.4289.1.S1_at	TCRA	T cell receptor, alpha	3.5
Bt.5336.1.A1_a_at	TF	transferrin	4.1
Bt.2017.1.S1_at	TFPI2	tissue factor pathway inhibitor 2	3.5
Bt.469.1.A1_at	TGFB1	transforming growth factor, beta 1	3.5
Bt.5401.1.S1_at	TGM2	transglutaminase 2 (C polypeptide, protein-glutamine-gamma-glutamyltransferase)	31.0
Bt.3011.1.A1_at	THBS3	thrombospondin 3	3.6
Bt.5136.1.S1_at	TIMP3	TIMP metallopeptidase inhibitor 3	3.7
Bt.26973.1.S1_at	TLL2	tolloid-like 2	-4.8
Bt.628.1.S1_at	TMED6	transmembrane emp24 protein transport domain containing 6	3.2
Bt.16326.1.S1_at	TMEM140	transmembrane protein 140	3.4
Bt.27029.1.S1_at	TMEM45B	transmembrane protein 45B	4.6
Bt.11061.1.S1_at	TNC	tenascin C	52.3
Bt.958.2.S1_at	TNFAIP6	tumor necrosis factor, alpha-induced protein 6	-3.5
Bt.24855.2.S1_at	TNFSF13B	tumor necrosis factor (ligand) superfamily, member 13b	3.2
Bt.5432.1.S1_at	TNIP1	TNFAIP3 interacting protein 1	5.5
Bt.5432.3.S1_at	TNIP1	TNFAIP3 interacting protein 1	4.9

Bt.8400.1.A1_at	TOX	thymocyte selection-associated high mobility group box	-4.1
Bt.4688.1.S1_a_at	TPCN1	two pore segment channel 1	3.4
Bt.15740.2.S1_at	TPD52L1	tumor protein D52-like 1	-7.2
Bt.15740.1.A1_at	TPD52L1	tumor protein D52-like 1	-6.5
Bt.24477.1.S1_a_at	TPST2	tyrosylprotein sulfotransferase 2	3.8
Bt.9083.1.S1_at	TRAF1	TNF receptor-associated factor 1	4.3
Bt.9208.1.S1_at	TREM1	triggering receptor expressed on myeloid cells 1	11.2
Bt.22857.1.S2_at	TRIB2	tribbles homolog 2 (Drosophila)	-5.7
Bt.18822.1.A1_at	TRIB2	tribbles homolog 2 (Drosophila)	-5.4
Bt.22857.1.S1_at	TRIB2	tribbles homolog 2 (Drosophila)	-5.7
Bt.22980.1.S1_at	TRIM21	tripartite motif-containing 21	3.2
Bt.24373.1.S1_at	TRIM47	tripartite motif-containing 47	5.4
Bt.22126.1.S1_at	TSPAN5	tetraspanin 5	-4.9
Bt.2294.1.S1_a_at	UBA7	ubiquitin-like modifier activating enzyme 7	3.2
Bt.5897.1.S1_at	UBD	ubiquitin D	17.1
Bt.5897.2.S1_at	UBD	UBD protein-like	7.1
Bt.2379.1.S1_at	UMPS	uridine monophosphate synthetase	4.4
Bt.20343.1.S1_at	UNC13D	unc-13 homolog D (C. elegans)	4.0
Bt.23233.2.S1_at	USP18	ubiquitin specific peptidase 18	3.7
Bt.23233.1.S1_at	USP18	ubiquitin specific peptidase 18	3.2
Bt.8962.3.S1_s_at	USP18	ubiquitin specific peptidase 18	3.7
Bt.7043.2.S1_a_at	VCAM1	vascular cell adhesion molecule 1	5.3
Bt.22506.2.A1_at	VIPR2	vasoactive intestinal peptide receptor 2	3.2
Bt.28243.2.S1_at	VNN1	vanin 1	-3.1
Bt.28243.1.S1_a_at	VNN1	vanin 1	-6.3
Bt.21876.1.A1_at	WNT11	wingless-type MMTV integration site family, member 11	3.2

Bt.5259.1.S1_at	XAF1	XIAP associated factor 1	6.8
Bt.5403.1.S1_at	XDH	xanthine dehydrogenase	4.3
Bt.23809.1.A1_s_at	XDH	xanthine dehydrogenase	5.2
Bt.18479.1.A1_at	ZNF608	Zinc finger protein 608	-4.6
Bt.1220.1.S1_at	---	---	17.7
Bt.22849.1.S1_at	---	---	11.3
Bt.15892.1.S1_at	---	---	13.2
Bt.22286.1.S1_at	---	---	3.6
Bt.29894.1.S1_at	---	---	3.8
Bt.6817.1.S1_at	---	---	4.9
Bt.4277.1.A1_at	---	---	-4.1
Bt.17725.1.A1_at	---	---	15.5
Bt.17489.2.S1_at	---	---	5.0
Bt.25182.1.A1_at	---	---	7.6
Bt.17489.1.A1_at	---	---	10.4
Bt.7140.1.S1_at	---	---	4.6
Bt.22498.1.A1_at	---	---	6.4
Bt.16114.1.S1_at	---	---	4.4
Bt.24214.1.S1_at	---	---	5.6
Bt.23977.1.A1_at	---	---	3.7
Bt.6817.2.S1_at	---	---	5.2
Bt.19826.1.A1_at	---	---	8.4
Bt.2501.1.S1_at	---	---	3.9
Bt.16538.2.A1_at	---	---	4.3
Bt.11081.1.S1_at	---	---	4.9

Bt.2997.1.A1_at	---	---	7.7
Bt.27127.1.A1_at	---	---	-4.7
Bt.20854.1.S1_at	---	---	3.4
Bt.20823.1.A1_at	---	---	4.9
Bt.6687.1.S1_at	---	---	4.1
Bt.17514.1.S1_at	---	---	5.5
Bt.2837.1.A1_at	---	---	5.8
Bt.18202.1.A1_at	---	---	3.2
Bt.18490.1.A1_at	---	---	5.5
Bt.25656.1.A1_at	---	---	5.3
Bt.24547.1.S1_at	---	---	5.9
Bt.9485.1.S1_at	---	---	6.0
Bt.9950.1.S1_at	---	---	5.3
Bt.12667.1.S1_at	---	---	7.2
Bt.12583.1.A1_at	---	---	7.0
Bt.17546.1.A1_at	---	---	8.1
Bt.25915.1.A1_at	---	---	-3.6
Bt.13965.1.S1_at	---	---	5.1
Bt.25196.1.A1_at	---	---	-6.4
Bt.27261.1.S1_at	---	---	4.5
Bt.16721.1.A1_at	---	---	4.5
Bt.26695.2.A1_at	---	---	3.8
Bt.17041.2.S1_at	---	---	3.8
Bt.26231.1.S1_at	---	---	4.9
Bt.2537.2.S1_at	---	---	3.2
Bt.21083.1.S1_at	---	---	-7.2

Bt.3452.1.S1_at	---	---	3.4
Bt.8015.3.A1_at	---	---	3.8
Bt.21929.1.S1_at	---	---	-3.2
Bt.25475.1.A1_at	---	---	-5.5
Bt.18444.1.A1_at	---	---	-4.2
Bt.23330.1.S1_at	---	---	-3.6
Bt.23148.1.S1_at	---	---	3.7
Bt.3138.1.S1_at	---	---	-6.3
Bt.16058.1.A1_at	---	---	8.1
Bt.23789.1.A1_at	---	---	3.5
Bt.28340.1.S1_at	---	---	3.1
Bt.984.1.S1_at	---	---	6.5
Bt.16538.1.S1_at	---	---	3.7
Bt.16058.2.S1_at	---	---	6.6
Bt.23275.1.S1_at	---	---	3.0
Bt.10150.1.S1_at	---	---	9.1
Bt.8724.1.S1_at	---	---	4.1
Bt.17090.1.S1_at	---	---	-3.2
Bt.20591.2.A1_at	---	---	4.7
Bt.21069.1.S1_at	---	---	3.5
Bt.24926.1.S1_at	---	---	-3.7
Bt.19567.1.A1_at	---	---	5.4
Bt.20591.1.S1_at	---	---	5.8
Bt.21102.1.S1_at	---	---	6.6
Bt.2501.2.A1_at	---	---	3.4
Bt.19567.2.S1_at	---	---	5.5

Bt.20295.1.A1_at	---	---	-6.0
Bt.13546.2.S1_at	---	---	5.6
Bt.13546.1.A1_at	---	---	4.8
Bt.8428.1.S1_at	---	---	4.1
Bt.17604.2.A1_at	---	---	3.4
Bt.12147.1.S1_at	---	---	5.0
Bt.12220.1.S1_a_at	---	---	3.1
Bt.17488.1.A1_at	---	---	3.9
Bt.20696.1.A1_at	---	---	-4.7
Bt.3072.2.S1_at	---	---	-4.0
Bt.7128.1.S1_at	---	---	-3.6
Bt.3072.1.A1_at	---	---	-4.0
Bt.24984.1.A1_at	---	---	5.3
Bt.17195.1.A1_at	---	---	6.4
Bt.19107.1.S1_at	---	---	3.3
Bt.17006.1.A1_at	---	---	3.4
Bt.24902.1.S1_at	---	---	-3.0
Bt.8264.1.A1_at	---	---	-4.4
Bt.8377.1.S1_at	---	---	-4.8
Bt.23509.2.A1_at	---	---	-5.4
Bt.22797.1.A1_at	---	---	-3.0
Bt.11427.1.A1_at	---	---	-3.9
Bt.17302.1.A1_at	---	---	-4.0
Bt.24940.1.A1_at	---	---	-4.6
Bt.27520.1.A1_at	---	---	-4.0
Bt.24426.1.A1_at	---	---	-8.3

Bt.28945.1.A1_at	---	---	-3.8
Bt.28855.1.A1_at	---	---	-5.4
Bt.3036.2.A1_at	---	---	-3.4
Bt.10130.1.S1_at	---	---	3.0
Bt.24587.1.A1_at	---	---	-4.9
Bt.24179.1.A1_at	---	---	-3.6
Bt.12609.1.A1_at	---	---	-4.5
Bt.16774.1.A1_at	---	---	4.4
Bt.17157.1.A1_at	---	---	-3.7
Bt.20507.1.S1_at	---	---	-3.4
Bt.21896.1.S1_at	---	---	-3.2
Bt.19792.1.A1_at	---	---	8.7
Bt.26299.1.A1_at	---	---	-3.4
Bt.15298.1.A1_at	---	---	-4.8
Bt.19957.1.A1_at	---	---	3.1
Bt.25303.1.A1_at	---	---	4.9
Bt.20580.1.S1_at	---	---	-4.1
